# 3CPET: finding co-factor complexes from ChIA-PET data using a hierarchical Dirichlet process

**DOI:** 10.1186/s13059-015-0851-6

**Published:** 2015-12-22

**Authors:** Mohamed Nadhir Djekidel, Zhengyu Liang, Qi Wang, Zhirui Hu, Guipeng Li, Yang Chen, Michael Q. Zhang

**Affiliations:** MOE Key Laboratory of Bioinformatics; Bioinformatics Division and Center for Synthetic & Systems Biology, TNLIST; Department of Automation, Tsinghua University, Beijing, 100084 China; Department of Molecular and Cell Biology, Center for Systems Biology, University of Texas, Dallas, 800 West Campbell Road, RL11, Richardson, TX 75080-3021 USA

**Keywords:** ChIA-PET, Chromatin interactions, Co-factors, Hierarchical Dirichlet process

## Abstract

Various efforts have been made to elucidate the cooperating proteins involved in maintaining chromatin interactions; however, many are still unknown. Here, we present 3CPET, a tool based on a non-parametric Bayesian approach, to infer the set of the most probable protein complexes involved in maintaining chromatin interactions and the regions that they may control, making it a valuable downstream analysis tool in chromatin conformation studies. 3CPET does so by combining data from ChIA-PET, transcription factor binding sites, and protein interactions. 3CPET results show biologically significant and accurate predictions when validated against experimental and simulation data.

**Electronic supplementary material:**

The online version of this article (doi:10.1186/s13059-015-0851-6) contains supplementary material, which is available to authorized users.

## Background

The expanding arsenal of techniques developed in the last decade to explore chromatin organization helped reveal the compartmentalized structures of chromatin [1–5]. These structures are established by complex processes that provide an environment for chromatin interactions and play an important role in juxtaposing regulatory elements to their target promoters [6, 7]. The establishment of long-range interactions plays a fundamental role in different cellular processes, such as the regulation of gene expression [7, 8] and the control of cell identity [9]. Additionally, it imposes constraints on the nuclear architecture, which influences replication timing [10] and genome maintenance [11].

Investigations into the mechanisms underlying chromatin loop formation showed that different protein complexes are involved in maintaining chromatin loop formation and stability [8, 12]. In the β-globin locus, Klf1, GATA1 and its co-factors FOG1 and Ldb1 play a key role in the formation of the locus control region (LCR)-promoter loop [13, 14]. Using a modified version of the Circularized Chromosome Conformation Capture (4C) method, namely, m4C-seq [15], researchers speculated that a collaboration between the key pluripotency transcription factors (TFs) (klf4, c-Myc, Sox2, and Esrrb) and known loop maintainer protein complexes (Mediator and Cohesion) is behind the establishment of the Nanog locus. In contrast, architectural proteins, such as CTCF, Cohesion, and Mediator [16, 17], demarcate themselves from other proteins by their wide involvement in shaping physical chromatin interactions.

The genome-wide analysis of the binding profiles of architectural proteins indicated that the combinatorial binding of co-factors contributes differently to chromatin organization [16–18]. Chromosome Conformation Capture Carbon Copy (5C) analysis of seven genomic loci around the key developmentally regulated genes in murine embryonic stem cells and neuronal precursor cells [16] shows that, within domains, different combinations between CTCF, Cohesion, and Mediator are established depending on the interaction length; the Cohesion–CTCF complex maintains intermediate-length interactions around tissue-specific genes, while the Cohesion–Mediator complex maintains interactions <100 kb. The respective knockdown of CTCF and Cohesion done by Zuin and his group [19] demonstrates that, even though CTCF and Cohesion are more likely to interact with each other, Cohesion is mainly involved in maintaining intra-topological domain interactions, while CTCF is important for their segregation.

Chromatin interaction analysis by paired-end tag sequencing (ChIA-PET) [4] is one of the methods for studying genome-wide interactions that involve a target protein. In the ChIA-PET method, DNA fragments associated with a protein of interest are first immunoprecipitated, then ligated to half-linkers, followed by a proximity-ligation step to connect adjacent DNA fragments. The resulting paired-end tags are then sent for sequencing and analysis to detect significant interactions. A key limitation of ChIA-PET and Chromosome Conformation Capture (3C) -based assays is that they can only give us some insight concerning DNA–DNA interactions but do not tell us much about the proteins that bring them together. Some candidate proteins can be inferred using chromatin immunoprecipitation sequencing (ChIP-seq) motif analysis, but it still presents a gloomy view of the gap in between.

Despite the development of biological methods to reveal the co-factors of a protein [20], we cannot make a clear distinction between proteins involved in establishing chromatin loops and those that do not. Thus, computational methods are still essential in predicting the proteins involved in the establishment of chromatin loops. However, until now, little attention has been paid from the computational side. Lan et al. [21] tried to integrate Hi-C and ChIP-seq data to infer the loop-maintaining protein network; however, the problem with their approach is that Hi-C contains many non-specific interactions, and they only inferred a single co-factor network that includes different types of transcriptional machinery (RNAP-II and RNAP-III); thus, it does not consider the specificity of TFs and nuclear foci.

Therefore, we present a new method, 3CPET, to fill this gap and try to associate co-factor proteins with the DNA–DNA interactions that they may maintain in an effort to help biologists identify loci-wise biomarkers. 3CPET is based on the observation that a protein can be a backbone element in some chromatin loops and dispensable in others [22]; thus, it tries to infer the set of the most probable co-factor complexes involved in maintaining the different spatial interaction contexts using the hierarchical Dirichlet latent allocation model [23].

## Results and discussion

### Inference of chromatin maintainer networks

The rationale behind 3CPET is that TFs can use a distinct combination of coactivators, depending on the genomic and spatial context. For example, studies of *Drosophila* showed that the pre-initiation complex (PIC) is assembled in a gene-dependent manner, where a PIC lacking TFIIB and D is used for histone genes [24] while different co-factors are recruited on genes encoding ribosomal proteins [25]. In the β-globin locus, GATA1 is known to maintain the LCR-loop formation [13, 14], while it does not play an important role in other loci. Here, we refer to each possible combination of co-factors and their interactions as the chromatin maintainer network (CMN), which participates in chromatin interactions and regulates several important biological processes such as gene transcription, DNA duplication etc.

**Fig. 1:**
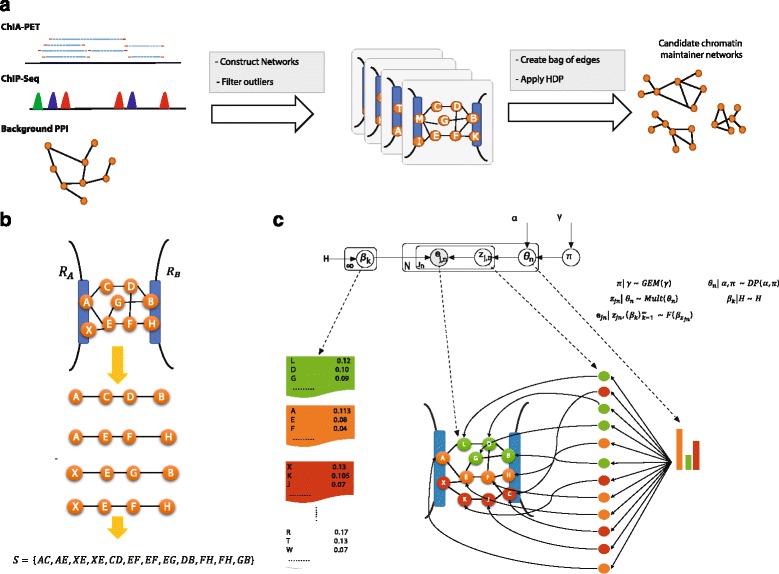
3CPET workflow. **a** 3CPET overflow: three types of data are provided, including DNA–DNA interactions (ChIA-PET), DNA–protein interactions (ChIP-seq), and PPIs. The algorithm builds a network connecting each DNA–DNA interaction, then passes them to the HDP model to infer the set of enriched networks. **b** Networks connecting DNA–DNA interactions are built by connecting each TF on one side to all of the TFs on the other side of the interaction. These networks are then converted into a bag of edges, where the frequency of each edge is equal to the number of shortest paths in which it appears. **c** The HDP model: each *β*_*k*_ represents a CMN and is a distribution over all possible edges. *θ*_*n*_ represents the CMN-per-network distribution. For each edge *e*_*jn*_, we associate a latent variable *z*_*jn*_ to indicate the membership to a CMN (*B*_*k*_). *z*_*jn*_ are drawn from the CMN-per-network distribution

Thus, to infer these sets of CMNs, 3CPET goes through the steps shown in Fig. 1a. First, we build, for each DNA–DNA interaction, a protein–protein interaction (PPI) network connecting the two interacting DNA regions. Thus, if we had 100 DNA–DNA interactions, 100 PPI networks would be built. We use this set of networks to infer the most enriched coactivator networks.

Given two interacting DNA regions, *R*_*A*_ and *R*_*B*_, proteins can be involved in establishing their interactions in two ways. The first way is by directly binding to the DNA, then by recruiting other proteins to interact with them to bring the two DNA sequences together. Thus, to build the protein networks connecting each DNA–DNA interaction, we first used ChIP-seq data to obtain the list of proteins involved at the boundaries of each chromatin interaction, and then we used a reference PPI to connect each TF in *R*_*A*_ to all of the TFs in *R*_*B*_ by considering the shortest path. Next, each network is converted into a bag of edges, where the frequency of an edge represents the number of short paths in which it participates, to capture important nodes in the network topology (Fig. 1b).

Because the publicly available PPI datasets represent a collection of results accumulated from different in vivo and in vitro experiments with different sources of noise and bias [26], a filtering step is needed. Therefore, only proteins known to localize to the nucleus and only interactions that show strong co-expression levels are considered. This step is very crucial for the accuracy of the final results because the direct use of the raw PPIs tends to introduce non-specific proteins, especially hub proteins.

Initially, 104,687 PPIs between 16,227 proteins were collected from the BioGRID database. We used this network in combination with gene expression and protein cellular location data to build two context-specific networks for the MCF7 and K562 cell lines, respectively. Hence, a reference network composed of 2714 proteins and 20,989 interactions was obtained for MCF7 and a network of 3144 proteins and 16,047 interactions for the K562 cells. The filtered network is used as a reference PPI for building the protein network collection that will be passed to the hierarchical Dirichlet process (HDP) algorithm to infer the CMNs. To avoid capturing general or non-significant TF interactions, we filtered out outlier interactions that appear in more than 80 % (maximum threshold) or less than 5 % (minimum threshold) of the built networks. The rationale behind this filtering step is that overrepresented interactions are likely to be artifacts resulting from shortest paths related to hub proteins or to be associated with some general proteins that do not bring too much information. The rare interactions, on the other hand, are some seldom or noisy interactions. More discussion about the effect of these thresholds is presented later in the manuscript. An R package incorporating all of these steps has been developed.

Based on the above assumptions and processes, the hierarchical Dirichlet latent allocation model [23] seems to be the most suitable model to infer (i) the number of CMNs and (ii) the proteins involved in each one. In a corpus of *N* DNA–DNA interactions, each linked by a network of *j*_*n*_ edges, our goal is to find the different groups of protein interactions that frequently appear together.

If we suppose that we have *K* CMNs, (*β*_*k*_)_*k* = 1_^*K*^, we may say that a DNA–DNA interaction is maintained by the CMN *β*_*k*_ if the majority of the edges of the network maintaining that DNA–DNA interaction belong to *β*_*k*_. Therefore, we can consider the set of protein interactions *e*_*j*,*n*_ connecting a chromatin interaction as a mixture of interactions sampled from different CMNs. We use the latent multinomial random variable *z*_*j*,*n*_ to indicate the CMN to which a protein interaction *e*_*j*,*n*_ belongs (Fig. 1c).

However, because we do not know how many CMNs exist, we allow the number to grow infinitely (*β*_*k*_)_*k* = 1_^∞^. Statistically, this scenario is equivalent to the problem of clustering the elements of grouped data while allowing the sharing of clusters between groups. This type of problem can generally be solved by applying a HDP model (supplementary methods in Additional file 1).

We used a Gibbs sampling approach, in which the number of CMNs is allowed to increase gradually, to infer the joint prior probability. After a sufficient number of iterations (in our case 1000), the algorithm converges to a steady state that allows us to infer (i) the number of CMNs, (ii) the edges constituting each CMN, and (iii) the probability for each DNA–DNA interaction to be maintained by the proteins of a CMN (see ‘Materials and methods’ and Additional file 1 for details). At the end of the algorithm, we build each CMN by selecting the top edges that capture a certain proportion of its cumulative distribution function (see Additional file 2 for the inferred CMNs used in this study). Discussion of the effect of the different thresholds is discussed later.

### Inference of ER-alpha associated co-factors and comparison with experimental data

ER-alpha is one of the extensively profiled TFs and plays an important role in breast cancer growth and progression [27]. Among all of the nuclear receptors, ER-alpha remains one of the main targets in tamoxifen-based therapy for breast cancer [28]. The elucidation of ER-alpha co-factors could enable the discovery of novel therapeutic targets for tamoxifen-resistant breast cancer [29].

Initial ER-alpha ChIP-seq profiling studies showed that ER-α preferentially binds to distal cis-regulatory elements away from the promoters of the regulated genes [28, 30] and recruits, in a temporal and spatial manner, a combination of collaborative factors to repress or activate its target genes [4, 20, 27, 31]. ChIA-PET analysis of ER-alpha associated factors [4, 31] also indicated that ER-alpha functions by establishing long-range interactions with the help of different collaborative factors. Among the well-known co-factors of ER-α are GATA3, FOXA1, LEF1, and RXRA [27]; however, many of these co-factors have been detected using protein–DNA binding assays.

**Fig. 2:**
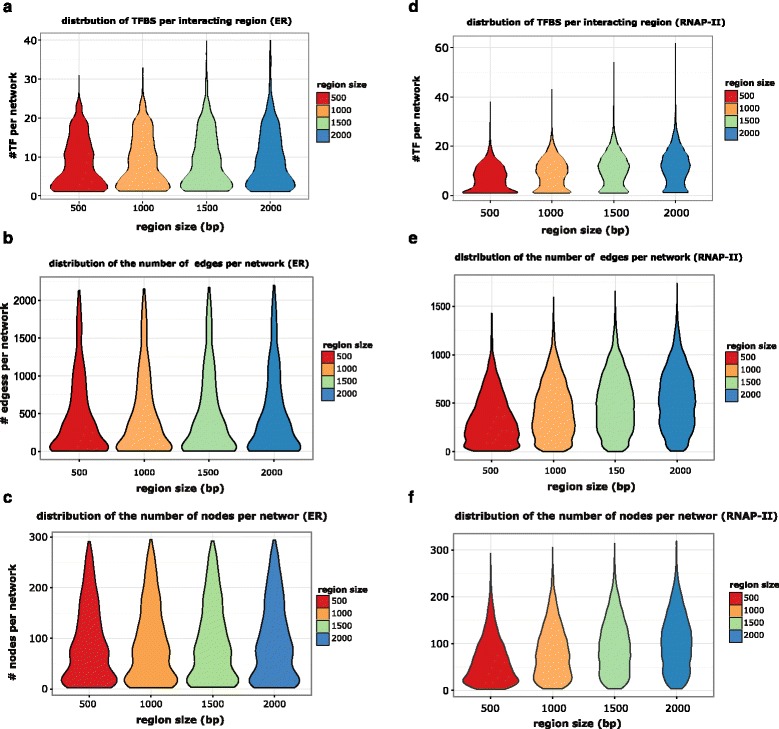
Constructed network statistics. **a** This violin plot shows the distribution of the number of TFBSs per interacting DNA region in the ER-alpha mediated interactions. Increasing the region size increases the number of overlapping TFBSs, but, on average, each region contains approximately nine TFs. **b** Distribution of the number of edges per network for ER-alpha associated interactions. **c** Distribution of the number of nodes per network for ER-alpha associated interactions. **d** Distribution of the number of TFBSs per region for RNAP-II associated interactions. On average, each region contains 11 TFs. **e** Distribution of the number of edges per network for RNAP-II associated interactions. The network sizes show a significant change when increasing the region size. **f** Distribution of the number of nodes per network for RNAP-II associated interactions. The number of nodes shows a significant change when changing the region size

Using ChIA-PET data published by Fullwood et al. [4], we generated a corpus of 1691 highly significant ER-alpha mediated long-range chromatin interactions in the MCF7 cell line (Additional file 3: Figure S1). We used these data in combination with ChIP-seq signals for 28 TFs (Additional file 4: Table S1). The MCF7-specific PPI network was constructed by filtering non co-expressed genes and proteins that do not locate at the nucleus. As the paired-end tags in ChIA-PET data tend to be more enriched in the middle of the interacting regions, we speculated that the TFs involved in maintaining chromatin interactions are also enriched near the center of these regions. Therefore, we only considered transcription factor binding sites (TFBSs) located 500, 750, 1000, 1500, and 2000 bp around the center of each region. The distribution of the number of TFs recruited per region size (Fig. 2a) indicates that, as expected, the number of recruited TFs will increase by increasing the region size, but, on average, the number of recruited TFs is stable. On average, nine TFs are recruited per region. We notice that the slight changes in TF recruitment do not have a large influence on the size of the constructed networks linking the DNA–DNA interactions (Fig. 2b, c). From these results, we can speculate that the set of ER-alpha collaborative factors is more or less restricted.

Depending on the region size, 3CPET inferred different numbers of CMNs with a mean of ten (Additional file 3: Figure S2). In the following analysis, we used the 1.5 kb related CMNs (due to their biological significance, discussed later).

As our local PPIs were generated by considering the shortest path in the PPI, we suspected that some overrepresented edges are artifacts of the PPI construction process that can be obtained regardless of ChIA-PET and ChIP-seq data. The background PPI bias effect is discussed in more detail in the following section. To assess the statistical significance of the obtained CMNs, we did a permutation test in which, for each iteration, we re-shuffled the TF binding sites, rebuilt the protein networks for each chromatin interaction using the same reference PPI, and re-inferred a new set of CMNs. The permutation test results obtained for the 1.5 kb regions show that two out of the ten CMNs failed to show their non-randomness (Additional file 3: Figure S3). This is because the nodes of these networks, on average, had a high connectivity in the reference network, which led to their appearance in many shortest paths as artifacts from the background PPI independently from the information included in ChIA-PET and ChIP-seq data. Meanwhile, the ER-alpha dataset contains 1691 interactions and about 200–500 DNA–DNA interactions will be filtered in the random shuffling step, which influences the accurate assessment of random interactions.

**Fig. 3:**
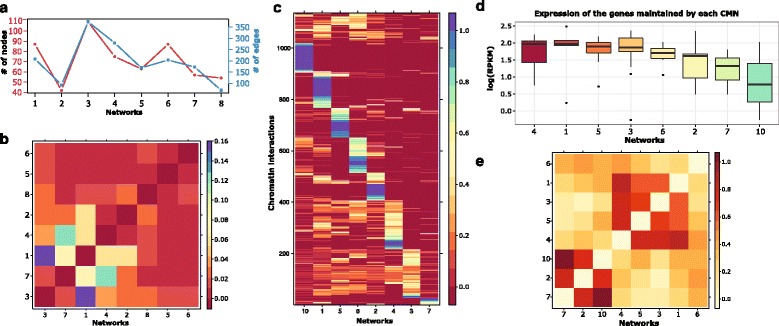
Characteristics of ER-alpha associated CMNs. **a** Plot showing the size of the different inferred ER-alpha associated CMNs. **b** Heat map showing the similarity between the CMNs. We notice a small degree of similarity, as the highest value is 16 %. **c** Heat map showing the degree of association of each interaction to a protein family. Each row is a ChIA-PET interaction and each column is a CMN. The blue color indicates little enrichment. The redder the color is, the more it is enriched. **d** Expression of the genes controlled by each CMN. Very distinct expression patterns are noticed for the different groups of controlled genes. **e** Heat map showing similarity between the expressions of the genes maintained by the different CMNs. *RPKM* reads per kilobase per million

The remaining eight significant networks tend to have different sizes, with the largest one having up to 110 proteins, mainly with elements known to be from the same protein complex (Fig. 3a). By checking the similarity between the different CMNs (Fig. 3b), we observe that the CMNs tend to share some core elements; however, they generally have heterogeneous structures, as the highest similarity is 16 %. These networks show a small-world distribution, with a large number of low-connected genes and a small number of hub proteins holding the interactions (Additional file 3: Figure S4).

We plotted the enrichment profiles of the eight CMNs in different ChIA-PET chromatin interactions (Fig. 3c). Notice that different chromatin interactions tend to be maintained by a specific network. By assessing the expression of the genes controlled by each CMN, we found that the expression of genes differs according to the co-factors involved. For example, CMN 4, which controls highly expressed genes, is mainly composed of transcription activation proteins (Additional file 3: Figure S5), such as enhancer-binding proteins CEPBA and P300, in addition to ER-alpha co-factors, such as FOXA1 and NR3C1. On the other hand, CMN 2, which controls genes with low expression, contains some transcription inhibition proteins mainly from the HDAC family (Fig. 3d, e).

**Fig. 4:**
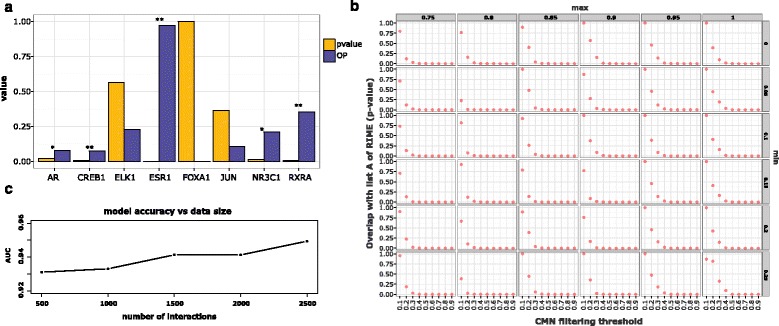
Biological validation. **a** Knockdown simulation results, in which we omitted ChIP-seq signals from ER-alpha (ESR1), FOXA1, and RXRA, and checked if 3CPET can predict the involvement of these proteins and can significantly recover the DNA interactions in which they may participate. The *x*-axis represents the TFs predicted by 3CPET not used in our dataset. The purple bars represent the observed proportion (OP) of the regions that actually contain the predicted proteins. The yellow bar represents the probability of obtaining similar enrichment in a random manner. Four out of seven predicted TFs were significantly enriched in the regions we claimed. **b** Tile plot showing the impact of the minimum and maximum thresholds using in filtering outlier interactions in the network corpus construction step, and the threshold used to build the CMNs. In each tile, we calculate the overlap *p* value between the list of predicted proteins and the proteins of list A in the RIME experiment. Overall, increasing the CMN construction threshold leads to better accuracy. Results that are more accurate can be obtained by filtering overrepresented interactions. **c** Accuracy of 3CPET results using simulated data. We simulated an interaction corpus of different sizes (500, 100, 1500, 2000, and 2500). We used 11 CMNs to sample a network for each interaction. The area under the curve (AUC) values were calculated using a multi-class receiver operating characteristic (ROC) analysis in which we checked whether 3CPET can truly re-assign interactions to their true CMN

To assess if 3CPET can significantly associate proteins with DNA regions, we simulated a knockdown experiment, in which we removed the signal of three important TFs: ER-alpha (ESR1), RXRA, and FOXA1. Then, we checked if 3CPET can predict their involvement in maintaining chromatin interactions and significantly associate them with chromatin interactions. We verified then whether the ChIP-seq signals of these TFs are significantly enriched in the regions we claim they are enriched in by doing a permutation test, in which we randomly pick an analogous number of chromatin interactions and check whether they show at least a similar enrichment value for the specific TF (see ‘Materials and methods’). Figure 4a shows that 3CPET was able to associate ESR1 and RXRA significantly with the DNA–DNA interaction they maintain; however, even if 3CPET was able to predict the involvement of FOXA1 in maintaining chromatin interactions, 3CPET failed to associate it significantly with the DNA–DNA interaction that it maintains. In addition to these factors, 3CPET was also able to predict the involvement of other TFs not initially included in our input signal, such as AR, ELK1, FOS, JUN, and NR3C1, in maintaining ER-alpha associated chromatin interactions. 3CPET was able to associate JUN and NR3C1 significantly with their predicted regions.

To detect ER-alpha non-DNA binding co-factors, Mohamed and his group [20] developed a mass-spectrometry-based method, rapid immunoprecipitation mass spectrometry of endogenous proteins (RIME), and used it to generate a list of ER-alpha interactors. We used the results generated by RIME in addition to some ChIP-seq data as ground truth to assess the reliability of our predictions. Two lists of ER-α collaborative factors were generated in the RIME experiment [20]: list A of 108 co-factors detected in three replicates and list B, which was less significant, of 250 co-factors detected in at least two replicates. We did a hyper-geometric test to test the significance of the overlap between these lists and the list of proteins inferred using different region sizes (Table 1). Mainly, the results show a significant overlap of the proteins in list A with the best results obtained for a region of size 1.5 kb (*p* = 2.334 × 10^-4^). Because it is impossible for our algorithm to infer proteins not in the reference PPI, we compared the overlap with only proteins present in our reference PPI. In this way, stronger overlap values were obtained, especially for the 1.5 kb region CMNs (2.0499 × 10^-10^ for list A) (Table 1). Many of the non-overlapping proteins had a degree of 1 in the reference PPI; thus, they were not members in many shortest paths. This indicates that there is a tradeoff in building the reference network where larger threshold values enable us to predict more candidate proteins with the penalty of introducing more noise.

**Table 1:** Overlap with RIME list A and list B

Flanking region size	A_ALL	B_ALL	A_PPI	B_PPI
500	7.1502E-04	0.7301	2.4248E-09	1.2201E-04
1000	3.5526E-04	0.6426	4.5359E-10	5.5431E-05
1500	2.3344E-04	0.5583	2.0499E-10	3.1794E-05
2000	3.3734E-04	0.5676	5.8403E-10	4.4591E-05

Many studies have been conducted to reveal some of the new drug targets that can give better recovery in breast cancer. Members of the peroxisome proliferator-activated receptor (PPAR) family have been revealed to be important biomarkers in different cancers including breast cancer. In this analysis, 3CPET was able to predict proteins such as PPARD, which has been shown to inhibit the growth of MCF7 cells [32].

### Sensitivity, robustness, and accuracy of 3CPET results

By considering the actual ER-alpha related CMNs and the results of the RIME experiment as ground truth, we assessed the effect of the different thresholds and of the input data on the different aspects of the 3CPET results. We studied the influence of three essential thresholds: the minimum and maximum thresholds used to filter outlier interactions in the local network construction step and the threshold used to select top edges used to build the CMNs. Basically, four types of analysis were performed.

The first analysis studied the effect induced by the different thresholds on the performance of 3CPET from two aspects. First, by checking the overlap with RIME data, a prediction result is deemed potentially useful if it has a more significant overlap with RIME data. Second, by measuring the degree bias of 3CPET results toward the background PPI, the extent to which a bad background PPI construction can affect our results can be checked.

The tile plot in Fig. 4b shows the effect of varying the three thresholds on the ability of 3CPET to predict RIME data significantly. We notice that regardless of the minimum and maximum values, the overlap significance with RIME data improves with increasing CMN construction thresholds, which is expected as more elements are introduced. However, if we compare the speed of convergence toward significant results between when we only filter overrepresented interactions (minimum = 0, top row) and when we only filter interactions with low frequency (maximum = 1, right column), we see that the filtering of overrepresented interactions leads to a faster convergence toward significant results, as the user needs to select about 40 % of the top edges to get a significant overlap, while they need to select about 50 % of the top edges to get significant results when only lowly represented interactions are removed. This indicates that overrepresented PPI interactions, which are generally associated with hub proteins (as they tend to attract many short paths) or are associated with general TFs, can be considered more as artifacts than real players in maintaining chromatin interactions. Of course, the importance of overrepresented interactions varies depending on how confident we are about the background PPI and how specific is the target protein used to generate ChIA-PET data. We may expect the co-factors of more general proteins, such as RNAP-II, to contain many artifacts compared to the more specific ones. Thus, users can adopt flexible thresholds when studying specific proteins and stricter ones when dealing with proteins that are more general.

The previous results imply the possible existence of a bias in 3CPET results toward hub proteins of the background PPI network. To check this assumption, we verified if the CMN proteins maintain a degree of connectivity similar to the background PPI. In other words, highly connected proteins are still highly connected in our results and vice versa. Thus, we calculated their degree of correlation in the CMN proteins and their degree in the background PPI (Additional file 3: Figure S6).

Overall, increasing the CMN construction threshold introduces more bias from the background PPI; however, a maximum correlation value of 0.5 is observed in the extreme case. This indicates that the background PPI construction step is very important in influencing the final results and the mere use of a public PPI without preprocessing can lead to false results. Cautious users can adopt a threshold of 40 % to 50 % to get a good balance.

To measure the performance of 3CPET quantitatively, we did a simulation analysis in which we generated 11 CMNs and used them to sample our input networks corpus. We checked the performance of 3CPET from two sides: its ability to predict the original CMNs and its ability to associate DNA–DNA interactions with their original CMNs. As the HDP inference is based on a Gibbs sampling process, the number of predicted CMNs differs in each run. Additional file 3: Figure S7 shows a case in which 3CPET successfully predicts all of the 11 CMNs and a case in which it predicts fewer CMNs (in this case, nine). We notice that in the latter case (Additional file 3: Figure S7b), the predicted CMNs constitute a mixture of the original ones; however, in the first case, all the 11 CMNs were recovered with a high degree of similarity (Additional file 3: Figure S7a). Amazingly, 3CPET accurately predicts the enrichment of the chromatin interaction to the original CMNs (Additional file 3: Figure S8). When the predicted CMN is a mixture, the associated CMNs are enriched in the chromatin interactions originally maintained by the mixture of the original CMNs.

To quantify this behavior, we calculated the accuracy of 3CPET in associating chromatin interactions with their original CMNs (see ‘Materials and methods’). Figure 4b shows the area under curve (AUC) values of the multi-class receiver operating characteristic (ROC) analysis given different data sizes. Notice that the increase in the number of chromatin interactions leads to predictions that are more accurate. This implies that, when more data are available and when the user carefully selects accurate inputs, 3CPET results can lead to predictions that are more useful.

The simulation results indicated that there is a certain influence of the library complexity on the final results. To investigate this question further, we simulated different library complexity values from the real data and checked its influence on the significance of the final 3CPET output. For ChIA-PET data, library complexity indicates the percentage of paired-end tags that lead to significant interactions discoveries. Thus, in our simulation, we suppose that the initial data is the full library and each time we sample a percentage from it. Ten samples were generated for each case. If we plot the distribution of the overlap probabilities with RIME data (Additional file 3: Figure S9), we notice that the overlaps get more significant with increasing experimental quality (more interactions detected) (Additional file 3: Figure S9a). However, low-quality experimental data leads to very variable results (Additional file 3: Figure S9b), but starting from 60 %, the variance gets tighter and even with 20 % complexity, fewer outlier results are obtained. This can be explained by the stability of the ChIP-seq signal, as the main ER-alpha core co-factor signal is highly enriched regardless of the number of discovered interactions (Additional file 3: Figure S10). Still, the fewer interactions are discovered, the more statistical power is lost and this opens the doors for more false positive discoveries. Consequently, if we plot the distribution of the number of the newly discovered proteins (the ones not included in the input ChIP-seq signal), we notice different runs on low-quality data can lead to very different results, while more stable results are obtained with an improvement in data quality (Additional file 3: Figure S11).

Another question that one may ask is how robust are the 3CPET results to input perturbations? To investigate this question, we run 3CPET separately on the ER-alpha ChIA-PET replicates and checked the similarity of the obtained CMNs to those predicted using just the common interactions. We clustered the predicted CMNs in each replicate according to their similarity to the CMNs in the common chromatin interactions (Additional file 3: Figure S12). The similarity to the CMN in the common interactions differs according to the used threshold; using minimum and maximum thresholds of 0.05 and 0.8, respectively, leads to very robust results with only one CMN of replicate 2 that showed no similarity to the common CMNs (Additional file 3: Figure S12a). However, when relaxing the maximum threshold to 0.95 and comparing with the common CMNs obtained using the same thresholds, we notice that many replicate CMNs failed to cluster with the common CMNs (Additional file 3: Figure S12b). The relaxing of the filtering thresholds enabled the introduction of more candidate proteins and increased the sensitivity of 3CPET to the input interactions. When the overlap between the replicates is not very high, it is advisable to run 3CPET on the common interactions and use tighter filtering thresholds.

To sum up, we can assert that overall 3CPET has a reasonable performance and shows stable behavior and sensitivity. A good selection of high-quality inputs have the potential to reveal new significant co-factor candidates.

### Controlling the number of CMNs and the sparsity of the results

The previously discussed minimum and maximum thresholds control the way in which the local PPI network corpus is prepared. However, once this corpus is passed to 3CPET, the final output depends on the behavior of the HDP algorithm. HDP is composed of three main layers. The first layer is the base measure *H* that constitutes the source of CMNs (*β*_*k*_)_*k* = 1_^∞^. The second layer is the global measure *G*_0_ that helps in sampling a discrete number of CMNs from H to be shared along the entire corpus. The third layer uses the probability measures *G*_*j*_, to select the appropriate CMNs for the edges of the *j*th local PPI from *G*_0_.

These three layers are controlled by three parameters *η*, *γ*, and *α*. The first parameter *η* is used to indicate our prior belief on the way the edges belong to CMNs. In other words, it shows how each *β*_*k*_ puts its weight on each edge. In our settings, this behavior is inherent to the Dirichlet distribution (Additional file 3: Figure S13). Smaller *η* values lead to a sparser edge-per-CMN distribution, while larger *η* leads to a more uniform one. The *γ* parameter is the concentration parameter used to control how *G*_0_ puts its mass on each CMNs. Smaller *γ* values favor the concentration of the mass on a small number of CMNs while larger *γ* values give almost similar preferences to all the CMNs. The *α* parameter also has a similar effect but on the local PPI level, with smaller *α* values favoring the edges of a PPI belonging to a small number of CMNs and larger *α* values allowing edges to be part of different CMNs.

Two criteria, sparsity and the number of topics, can mainly be used to describe the impact of the HDP parameters on the final results. Sparsity enables users to control the number of CMNs allowed to maintain a DNA–DNA interaction and how CMNs are allowed to share common edges. By combining the results from Additional file 3: Figures S14 and S15, we notice that *η* is the main player in controlling the sparsity, whether on the edge-to-CMN level or the PPI-to-CMN level, with higher values of *η* leading to less sparse results. Additionally, we observe that *γ* values larger than 1 compared to values smaller or equal to 1, enable the local PPI networks to have fewer CMNs (Additional file 3: Figure S15), and at the same time, leading to a sparser edge-to-CMN distribution (Additional file 3: Figure S14). For fixed *η* and *γ*, increasing *α* helps slightly in decreasing the sparsity. *γ* = 0.01 is a special case because, as we will see later, there were only one or two CMNs, and thus all the edges had a certain probability of belonging to them.

In fact, the sparsity levels are the results of the increase in the number of CMNs. As we can see in Additional file 3: Figure S16, *γ* values larger than 1 lead to an increase in the number of CMNs per DNA–DNA interaction. When combined with smaller *η* values, CMNs will have their mass concentrated on a small number of edges, which leads to a higher sparsity as shown in Additional file 3: Figure S15. From Additional file 3: Figure S16, we notice also that an increase in *η* values leads to a decrease in the number of clusters. We notice the same trend when varying *α* with fixed *η* and *γ*. These trends can be understood by examining the sampling scheme shown in the supplementary method in Additional file 1.

In our case, we used *η* = 0.01 to enable edge-to-CMNs sparsity, and *γ* = *α* = 1, to give an unequal probability to all CMNs to control DNA–DNA interactions. However, users can tune these parameters according to their previous knowledge about the protein of interest. For very specific proteins, maybe the users will be interested in having a small number of CMNs, while for a general protein, the user can allow more CMNs to be detected to increase the granularity.

### RNAP-II chromatin maintainer networks

Transcription is a complex dynamic process that relies on different proteins. The elucidation of proteins and TFs interacting with the transcriptional machinery can help us to make a step forward in understanding the transcriptional regulome. To explore the protein apparatus recruited by the transcriptional machinery to maintain chromatin interactions, we used K562 ChIA-PET interactions with RNAP-II as bait [33]. As the number of common interactions between the RNAP-II replicates (30,396) is about three times the number of common interactions for ER-alpha (3019), we had the opportunity to use a tighter filter value (≥5) to generate a corpus of 17,253 DNA–DNA interactions (Additional file 3: Figure S17). Like the ER-alpha workflow, we only considered co-expressed proteins known to localize at the nucleus. In addition, we used the ChIP-seq signal of 37 TFs (Additional file 4: Table S2).

From Fig. 2d, we can see that, in contrast to ER-alpha, the number of TFs binding to the interacting DNA regions shows a more concentrated distribution profile with the majority of the interactions bound by the 11 TFs. Meanwhile, for ER-α, a more broad distribution is present. However, the size of the networks connecting the DNA–DNA interactions shows a significant change between the different region sizes (Fig. 2e, f). Thus is because the RNAP-II background co-expression PPI is more connected than the ER-alpha one. Thus, the length of the shortest paths connecting two DNA fragments is shorter in the RNAP-II network, which is the opposite of ER-α, where the connection of two DNA fragments needs more proteins, leading to bigger networks.

**Fig. 5:**
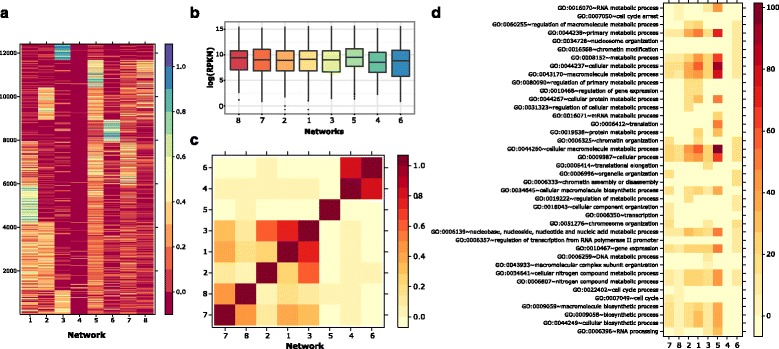
Characteristics of RNAP-II associated CMNs. **a** Heat map showing the degree of association of each interaction to a protein family. Each row is a ChIA-PET interaction and each column is a CMG. The blue color indicates little enrichment. The redder the color is, the more it is enriched. **b** Expression of the genes controlled by each CMN. **c** The clustering of the expression of genes per CMN indicates that the genes controlled by CMNs 4, 5, and 6 demarcate themselves from the others. **d** Gene ontology enrichment analysis of the genes controlled by the different RNAP-II associated CMNs. MNN 5 tends to control mRNA metabolite process related genes, while 6 and 4 maintain genes related to chromatin assembly. Genes controlled by CMNs 1, 2, and 3 tend to be more general, and the ones controlled by 7 and 8 are mainly involved in the cell cycle. *RPKM* reads per kilobase per million

We used regions of size 1500 bp around the center of DNA interactions for the downstream analysis because they represent the typical enhancer size. By applying 3CPET, eight CMNs were obtained (Fig. 5a). Many of the transcription-related proteins were predicted to be involved in the maintenance of chromatin interactions, such as CREBBP, which is known to play the role of a scaffold in stabilizing transcriptions, the enhancer-associated protein P300, and some of the Mediator complex proteins, such as MED1, in addition to architectural proteins, such as CTCF and RAD2 [34–36].

Analysis of curated human protein complexes from the CRUM database [37] shows that all inferred CMNs contain elements of the RNAP-II core complex, with CMNs 1, 2, and 4 more enriched for chromatin remodeling complexes, such as SWI/SNF and BAF, while the others are more enriched for the PIC-related proteins, with CMNs 7 and 8 containing a TATA-binding protein-free TAF-containing complex.

To estimate the number of true predictions, we checked if the ChIP-seq signal of some predicted proteins is significantly enriched in the regions claimed by 3CPET in Fig. 5a. Therefore, we used the ChIP-seq signal of 13 proteins, for which published data were available but not included in our input dataset. Among these 13 ChIP-seq signals are some proteins known to maintain chromatin interactions, such as CTCF and P300. Eleven out of the 13 predicted TFs showed a significant enrichment in the claimed regions (Additional file 3: Figure S18). Mainly, the predicted proteins showed high abundance, with 64 % of the real signal located in the predicted regions.

To assess if the regions enriched for each network encoded different functionality, we considered the genes located 2.5 kb around the center of the interaction regions with more than 50 % enrichment for a specific CMN and compared their gene ontology (GO) annotation (Fig. 5d). The correlation between the gene expressions of the genes maintained by each network showed that groups of CMNs control different co-expressed genes (Fig. 5b, c), with networks 7 and 8 maintaining co-expressed genes involved mainly in cell-cycle, transcription, and chromatin regulation (Fig. 5d). CMNs 2, 1, and 3 controlled co-expressed genes involved in metabolic process regulation. CMNs 4 and 6 regulated genes related to chromatin assembly. However, CMN 5, mainly composed of SWI/SNF elements, controlled translation and mRNA metabolite process related genes and showed a higher expression profile compared with the others (Fig. 5b, c, and d).

Compared with the ER-alpha gene regulation results, the expression profile of the genes controlled by the RNAP-II related CMNs does not show a very visible variation between the different regions, which indicates that some co-factors play a substitutable role and do not have a large influence on the expression of genes involved in chromatin interactions. However, for ER-α, the results show that different combinatorial recruitment patterns have an impact on the way genes are regulated.

The *β*-globin locus is one of the most intensively studied loci in hematopoietic cells. With the development of 3C-based methods, it has been used as a model system in understanding the mechanisms of chromatin loop establishment [13, 14, 38, 39]. *β*-globin is a multi-gene locus composed of several globin genes and is regulated through regulatory elements located mainly at the LCR [40] through loop formation. Several proteins have been shown to be involved in LCR *β*-globin loop formation, such as GATA1, LMO2, and CTCF.

In our dataset, two *β*-globin loops were enriched. The outer loop connects the LCR to the region between the *A*_*γ*_ and *δ* genes, and an inner loop connects the ε gene to the *G*_*γ*_ gene (Additional file 3: Figure S19). 3CPET predicted the enrichment of two networks in this region, with the outer loop showing an enrichment for the RNAP-II and CTCF related network (CMN 3) and the network involving the GATA1 and P300 network (CMN 1). Meanwhile, the inner loop showed high enrichment for the transcription-related network (CMN 3). The results of literature mining show the enrichments of these two networks for both terms *β*-globin and RNAP-II (Additional file 4: Table S3).

### 3CPET as an R package

**Fig. 6:**
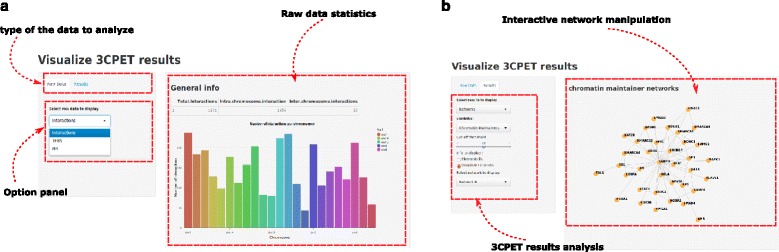
Results produced by the R3CPET package. **a** Screenshot showing an example of the web interface of the 3CPET package for raw data analysis. **b** Screenshot showing an example of the web interface of the 3CPET package for results analysis

To facilitate the execution and analysis of the data using the proposed method, we implemented 3CPET as an R package (Fig. 6, Additional file 5). The package has three main functionalities:

1. It contains functions to manipulate and load ChIA-PET interactions, ChIP-seq signals, and the PPI.
2. In addition, it has methods to run the HDP model and cluster the DNA interactions according to their enrichment profiles, as well as the ability to perform GO and expression analysis.
3. The package also has a set of functions to visualize and generate different plots in the R environment or interactively using a web interface based on Shiny.

Using the 3CPET web interface (Fig. 6a), users can display statistics related to the raw data, the number of ChIA-PET regions per chromosome, and the TF abundance distribution per region. Users also can analyze 3CPET results (Fig. 6b), where they can interact with the inferred networks, and display their occupancy profile and the DNA regions maintained by each network. R3CPET is available under a GPL (≥2) license at: http://www.bioconductor.org/packages/release/bioc/html/R3CPET.html.

The time and space complexity depends on many factors, such as the number of Gibbs iterations, the sparsity of the results, and the number of topics. In Additional file 3: Figure S20, we plotted the memory occupancy of R3CPET when running the HDP algorithm on ER-alpha data. We notice that, overall, 3CPET occupies 94–97 Mb, which is a reasonable amount. Increasing *η* leads to a slight increase in memory occupancy as the data are less sparse (Additional file 3: Figures S14 and S15). From the execution time perspective, we notice that there is not a fixed pattern, but overall larger *η* values lead to longer execution times, which is mainly due to the reduced sparsity of the internal structures (Additional file 3: Figure S21). One may expect that the increase of the number of inferred CMNs has an influence on the execution time; however, from Additional file 3: Figure S22, we notice that there is no clear correlation between the number of inferred CMNs and execution time, which indicates that sparsity is the main player in controlling the execution time. Overall, it takes 3CPET from 20 to 30 min to calculate the final results; the fastest case is about 5 min.

As expected, the memory and time requirements for 3CPET gradually increase with the DNA–DNA corpus size. The plots in Additional file 3: Figure S23 clearly indicate a linear scaling of its time and memory requirements. The results shown in Additional file 3: Figure S23 show the trends obtained using the HDP parameters *η* = 0.01 and *γ* = *α* = 1, but similar requirements for data size are observed for different parameter values.

## Conclusions

We have developed 3CPET, a tool based on a non-parametric Bayesian approach, to infer the set of the most probable protein networks that could be involved in maintaining chromatin interactions, making it a valuable downstream analysis tool in chromatin conformation studies. Our results show that a protein can be a backbone element in establishing some interactions and disposable in others. We also showed that the combinatorial recruitment of co-factors can have a large influence on the expression of the regulated genes, such as for ER-alpha, while it can also maintain the same functionalities with a smaller influence on the way in which regulated genes are expressed, as for RNAP-II.

3CPET exploits a HDP, which has the advantage of non-parametrically inferring (i) the number of distinct co-factor networks involved in maintaining chromatin interactions and (ii) the set of proteins in each network. Although the inference can be done using different clustering methods, the most widely used methods require a pre-existing knowledge of the number of networks or put certain assumptions on the distribution of the data.

We tested our approach for chromatin interactions associated with both ER-alpha and RNAP-II. For ER-alpha, 3CPET was able to predict many of the well-known co-factors, such as FOXA1, RXRA, NCOR1, and KLF1, among others, and showed a significant overlap with the RIME experiment results. The analysis of RNAP-II related interactions also enabled us to predict many proteins known to be involved in transcriptional machinery and enhancer binding. In both analyses, 3CPET was able to predict architectural proteins, such as CTCF and Cohesion.

We also performed a different type of analysis to investigate the performance and stability of our approach. We investigated the influence of the different thresholds on the input to 3CPET and we ran a simulation and robustness analysis to evaluate the accuracy and the stability of our method. Conjointly, we performed a comprehensive study on the influence of the HDP algorithm parameters on the number of inferred CMNs and the sparsity of the assignments to give general guidance to users. We showed that smaller HDP parameter values are more suitable in the study of specific proteins while larger values can be used to study general proteins. In addition, we did a basic time and memory profiling study of 3CPET to give an idea of the conditions affecting its time and memory requirements.

Overall, 3CPET showed reasonable performance and good stability. However, due to the dynamic nature of the protein interactions, we do not claim that the elements of the inferred networks are involved in maintaining the interactions at the same time, as some of them may take part in different stages of the transcription regulation process and others are involved in different stimulation conditions.

## Materials and methods

### Gene expression data

Gene expression data were downloaded from the GEO repository. For the MCF7 cell line, we used the dataset GSE848 [41], which contains a collection of 30 time-course microarray samples on which MCF7 cells were stimulated using different selective estrogen receptor modulators, such as raloxifene and estradiol. For K562 cell lines, we used the GSE11670 [42] dataset, which contains six time-course samples for ICL670-treated K562 cells.

### ChIA-PET data

For the MCF7 cell lines, we used common interactions between replicates 1 and 2 from the processed ChIA-PET data in the GEO dataset GSM970212 [4]. We used common ChIA-PET interactions [36] (E-GEOD-33664) between the two ChIA-PET replicates, with saturated Pol-II for the K562 cell lines. The data for both K562 and MCF7 were processed, by the original authors, using ChIA-PET tools [43] that use a hyper-geometric model to handle systematic bias and detect significant interactions.

### ChIP-seq data

We used the available TF ChIP-seq data signal (Additional file 4: Table S1) in the ENCODE project for the MCF7 cell lines [44]. For the K562 cell lines, we used 37 ChIP-seq signals from the ChIPBase database [45], which contains ChIP-seq peaks from different experiments, including ENCODE. The other 13 ChIP-seq signals used for validation were downloaded from the ENCODE project. We only considered common peaks for signals with more than one replicate or experiment results in the same cell line (K562 or MCF7).

### Reference PPI construction

We built the reference PPI by combining physical PPI information from the BioGRID database and an estimated co-expression network. First, the co-expression expression network was built using the weighted gene co-expression network analysis method, which tries to build a small-world co-expression network by raising the absolute value of the correlation between genes to a power *β* (soft threshold) until a good fit is found [46]. We used a soft threshold of 5 for MCF7 and 6 for K562 to obtain a small-world co-expression network (Additional file 3: Figure S23).

After calculating the weights, we calculated a topological overlap index that indicated the topological proximity between edges, given the weights obtained using the soft threshold. This index enables us to detect isolated nodes and nodes with very limited connectivity. In our case, we removed all nodes that show connectivity less than 50 % of that for all nodes. The use of the median here as a threshold gives more stable results than the use of the mean or other descriptive statistics. Finally, an unweighted adjacency matrix was used to build the co-expression network. As our aim is to capture physical interactions, we used the co-expression network to filter out the BioGRID PPI network to keep only co-expressed connections.

### Chromatin maintainer network inference

A hierarchical Dirichlet model is suitable for classification problems in which data are organized into groups and the elements of each group constitute a mixture of clusters. In our case, local PPI networks are groups of edges composed of a mixture of CMNs and we want the CMNs to be shared across the local PPI. HDPs have been widely used in document classification, where the words of each document are seen as a mixture of words sampled from different topics (for example, a bioinformatics document is a mixture of biology, statistics, and programming in different proportions). The aim of using HDP is to find the number of clusters in a given data corpus and the mixing proportions in each group while allowing the sharing of these clusters between groups (for example, group 1 can have elements from cluster 1 and also group 2).

As we do not know how many clusters are in our corpus of networks, we suppose that this number can grow to infinity (*β*_*k*_)_*k* = 1_^∞^ and we will suppose that they are sampled from a continuous base distribution *H*. The mere sampling of the cluster atoms *β*_*k*_ from *H* cannot guarantee that two chromatin interactions are maintained by the same CMN, as the probability of sampling the same CMN again from *H* is very low. Thus, the HDP algorithm introduces another layer in which we sample a discrete number of atoms into the distribution *G*_0_ from which the other chromatin interactions can sample their corresponding CMNs, which allow the sharing of CMNs, as shown for example in Fig. 3c (for detailed mathematical formulas, check the supplementary methods in Additional file 1).

In our case, we used the HDP code published by the original author and integrated it into our R3CPET package with some minor modifications [23]. In this implementation, the burn-in period depends on the data size. Here the burn-in is done in one Gibbs sweep with re-sampling after processing ten local PPI networks. Therefore, for ER-alpha, the burn-in period was more than 100 samples and for RNAP-II, the burn-in step consisted of more than 1500 samples. After the burn-in period, the inferred CMNs were reported after 1000 Gibbs-sampling sweeps.

### Check the significant enrichment of the inferred TFs

To check if 3CPET can accurately predict the binding of TFs to chromatin interactions, we used some TFs as a control. Thus, we did not include their ChIP-seq peak in the input of 3CPET and we checked if they significantly bind to the regions claimed by 3CPET.

Let TF_*i*_ be a TF predicted by 3CPET and let *N* be the number of interactions we claimed to be controlled by the networks in which TF_*i*_ participates. Let ${S}_{{\mathrm{TF}}_i}$ be the real ChIP-seq signal of TF_*i*_; then, the observed proportion of interactions truly bound by TF_*i*_ can be calculated as $\mathrm{OP}=\frac{\left|{S}_{{\mathrm{TF}}_i}\cap N\right|}{N}$ where | . | is the number of interactions interacting with the signal.

To check if this proportion is signify enriched than would be expected by chance, we generated a null distribution in which we randomly select *N* interactions from our ChIA-PET data 500 times and calculate the probability of observing an enrichment larger than the observed OP.

### CMN similarity

As described in the HDP process (see additional methods in Additional file 1), each CMN represents a distribution over the possible edges in our corpus. Thus, comparing two CMNs is similar to comparing two distributions. In our analysis, we used the Jensen–Shannon diversion to calculate the similarity:

$$ \mathrm{s}\mathrm{i}\mathrm{m}\left(p,q\right)=1-\mathrm{J}\mathrm{S}\left(p,q\right) $$

and

$$ \mathrm{J}\mathrm{S}\left(p,q\right)=\frac{1}{2}\;\left[\;D\left(p,\;\left(p+q\right)/2\right)+D\left(q,\;\left(p+q\right)/2\right)\;\right] $$

where $D\left(p,q\right)={\displaystyle {\sum}_{i=1}^T{p}_{e_i}\; \log \left({p}_{e_i}/{q}_{e_i}\right)}$ and *T* is the total number of different edges in our corpus.

### CMN validation: permutation test

To check if the obtained CMNs are generated due to the input data or due to the influence of the background PPI, we perturbed the input data by randomizing the existing chromatin interactions and the binding positions of the TFs, then fed them to 3CPET while keeping the background PPI intact. The significance is then assessed by comparing the degree of overlap of the predicted CMNs to the randomly interfered ones. This design enable us to detect the CMNs that were constructed due to the influence of hub nodes in the background PPI as they tend to attract the short paths toward them. We calculated the *p* value as follows.

Let *N*_*i*_ be a CMN with *n*_*i*_ edges and let *N*_*i*_^'^ be a CMN with *n*_*i*_^'^ edges inferred from the random input. We check if they significantly overlap as follows.

Let *M* be the number of nodes in *N*_*i*_ ∪ *N*_*i*_^'^. The number of possible edges is

$$ E=\frac{M\;\left(M-1\right)}{2}. $$

The number of common edges between *N*_*i*_ and *N*_*i*_^'^ is *c*.

Then, for each iteration the *p* value is equal to, we obtain *c* common edges by randomly taking two groups of *n*_*i*_ edges from the complete graph. The general formula can be summarized as follows:

$$ P={\displaystyle \sum_{i=c}^{\min \left\{E\left({N}_i\right),\;E\left({N}_i^{\hbox{'}}\right)\right\}}}\frac{C_{E\left({N}_i\right)\;}^i\times {C}_E^{E\left({N}_i^{\hbox{'}}\right)-i}}{C_E^{\;E\left({N}_i^{\hbox{'}}\right)}} $$

where *E*(.) indicates the number of edges in the network.

After 1000 iterations, we correct the *p* values using the *statmod* package in R.

### Simulation

The simulation process consisted of generating a corpus of networks and their enrichment profiles on the ChIA-PET data. We used the 11 CMNs predicted by 3CPET using minimum and maximum thresholds of 0.05 and 0.9 to generate the network corpus. For each chromatin interaction, we sample the CMN profile associated with it, then we use these proportions to sample edges from the 11 CMNs by first sampling an indicator variable *z*_*i*_ then sampling an edge from the CMN ${\beta}_{z_i}$. The size of the networks was uniformly sampled between 10 and 200 edges.

In the examples in Additional file 3: Figures S7 and S8, we sampled edges only from the CMN with the highest proportion to get a heat map that can be visually compared.

### Multi-class ROC analysis

To estimate the accuracy of 3CPET predictions, we did a multi-class ROC analysis in which we checked if 3CPET can accurately assign chromatin interactions to their corresponding CMNs. Therefore, for each chromatin interaction, we assigned a label *l*_*i*_ that indicates the CMN associated with it. As the number of CMNs differs each time we run 3CPET due to the Gibbs sampling process, some of the estimated CMNs can represent a mixture of two original CMNs. Hence, we defined a mapping function *f* between the original (*CMN*_*k*_)_*k* = 1_^11^ and the inferred ones (*CMN*_*k*_^'^)_*k*_^*K*^ as

$$ f(k)= \arg \underset{x}{ \max }\ \mathrm{s}\mathrm{i}\mathrm{m}\left({\mathrm{CMN}}_k,\;{\mathrm{CMN}}_x\hbox{'}\right),\kern1.12em x=1,\dots, K. $$

We hypothesized that if a CMNx' showed a similarity to an original CMNk, then they should also maintain the same chromatin interactions. Consequently, if CMNx' is a mixture of two original CMNs, than it should also maintain their corresponding chromatin interactions. Hence, our multi-class ROC analysis can literally be expressed as

$$ \mathrm{A}\mathrm{U}\mathrm{C}=\mathrm{multi}-\mathrm{class}\ \mathrm{ROC}\left(\;f\left({\mathrm{labels}}_{\mathrm{original}}\right),\;{\mathrm{labels}}_{\mathrm{predicted}}\right). $$

In the plot in Fig. 4c, for each data size, we calculate the mean AUC after 100 simulations.

### Literature mining

To assess if a network is significantly enriched for a certain concept, we first used the literature mining method in [47] to construct a concept-related gene co-occurrence network *G*. Let *P* be the set of all proteins in the union of our 11 networks, *TP* = *P* ∩ *G* be the number of proteins annotated for this particular concept, *c*_*i*_ = *V*_*i*_ ∩ *G* be the number of proteins in network *V*_*i*_ that exist also in *G*. Then, the *p* value for getting more *c*_*i*_ proteins related to the concept from a set of *P* proteins having *TP* of them annotated for the concept is calculated as

$$ P={\displaystyle {\sum}_{k={c}_i}^{TP}}\frac{C_{TP\;}^k\times {C}_{\left|P\right|-TP}^{\left|{V}_i\right|-k}}{C_{\left|P\right|}^{\left|{V}_i\right|}} $$

where | . | represents the size of the set.

The *p* values were then corrected using a Benjamini–Hochberg test.

### 3CPET R package

The 3CPET method has been implemented as an R package under the name R3CPET. R3CPET is available at Bioconductor (http://www.bioconductor.org/packages/release/bioc/html/R3CPET.html) under a GPL (≥2) license. The source code is also available at Bioconductor and our Git repository at https://github.com/sirusb/R3CPET.

### Ethical considerations

The competent research ethics committee confirmed that no ethical approval was needed.

## Additional files

Additional file 1:
**Additional methods.** (DOCX 30 kb) Additional file 2:
**Excel file listing the inferred ER-alpha and RNAP-II related networks.** (XLSX 35 kb) Additional file 3:
**All supplementary figures (S1 to S23) and their corresponding legends.** (DOCX 6465 kb) Additional file 4:
**All supplementary tables (S1 to S3) and their corresponding legends.** (DOCX 15 kb) Additional file 5:
**3CPET package manual.** (PDF 976 kb)

## Abbreviations

AUC: area under the curve
ChIA-PET: chromatin interaction analysis by paired-end tag sequencing
ChIP-seq: chromatin immunoprecipitation sequencing
CMN: chromatin maintainer network
GO: gene ontology
HDP: hierarchical Dirichlet process
LCR: locus control region
PIC: pre-initiation complex
RIME: rapid immunoprecipitation mass spectrometry of endogenous proteins
ROC: receiver operating characteristic
TF: transcription factor
TFBS: transcription factor binding site
