# Supplementary material for: 3CPET: finding co-factor complexes from ChIA-PET data using a hierarchical Dirichlet process
Source: Genome Biol. 2015 Dec 22;16:288. doi: 10.1186/s13059-015-0851-6 (PMC4716632; doi:10.1186/s13059-015-0851-6)
Supplement: Additional file 1: — Additional methods. (DOCX 30 kb) [file 13059_2015_851_MOESM1_ESM.docx]

# Supplementary Methods for :

# 3CPET: Finding Co-factor Complexes from ChIA-PET data by using Hierarchical Dirichlet Process

**Mohamed Nadhir Djekidel1,** **Zhengyu Liang^1^, Qi Wang^1^, Zhirui Hu^1^, Guipeng Li^1^, Yang Chen^1^**^§^**, Michael Q. Zhang^1,2^** ^§^

^1^ MOE Key Laboratory of Bioinformatics and Bioinformatics Div, Center for Synthetic and System Biology, TNLIST /Department of Automation, Tsinghua University, Beijing 100084, China;

^2^ Department of Molecular and Cell Biology, Center for Systems Biology, The University of Texas, Dallas 800 West Campbell Road, RL11 Richardson, TX 75080-3021, USA

**The Hierarchical Dirichlet process**

In corpus of grouped data we suppose that the elements of each group represent a mixture of different clusters and that the groups share clusters between them. Let each group $k$ be represented by the variable $\beta_{k}$ that we call the atom of the cluster. We suppose that the atoms of the clusters $\left( \beta_{k} \right)_{k=1}^{\infty}$ are sampled from a continuous distribution $H$ (in our case a symmetric Dirichlet distribution with parameter $\eta$) . However, as the direct sampling from H doesn’t guaranty the sharing of clusters between groups (as it hard to get the same sample more than once) a second discrete distribution $G_{0}$ sampled from $H$ is used to sample cluster atoms from it. Here, each $\beta_{k}$ represents a chromatin maintainer network.

If we consider the set of edges that appear in our corpus of networks as our vocabulary, we can notice each $\beta_{k}$ (CMN ) represent a distribution over edges in the vocabulary. This concept of sampling a distribution from distribution measures can be expressed by a Dirichlet Process (DP).

Let $G_{n}$ be the measure associated with each network distributed according to a group specific DP. $G_{n}$ can then be expressed as $G_{n}=\sum_{k=1}^{\infty} \alpha_{k} \delta_{\beta_{k}}$, where $\beta_{k}$ are the atoms sampled from $G_{0}$ and $\alpha_{k}$ is the concentration parameter. However, to allow the sharing of atoms between networks, $G_{0}$ itself is drawn from a DP with base distribution $H$ and concentration parameter$\gamma$ [[1](#_ENREF_1)]. For each network in our corpus, we sample a multinomial probability vector $\theta_{n}$ that indicate the enrichment of a given CMN in that interaction. Given $\theta_{n}$ we can sample the group indicators $z_{jn}$ for each protein interaction$e_{jn}$ in the network linking the edges of the chromatin interaction. The aim then is to infer the values of the different lattent variables in the model and thus inferring the number of CMNs and the regions that they maintain.

The posterior distributions are calculated using a Gibbs sampler by considering the finite model as derived in [[1](#_ENREF_1)] (Posterior Sampling by Direct Assignment approach). Briefly, in a finite setting in which we have $L$ mixture components where only $K$ are currently used, the model can be expressed as:

$\pi| \gamma\sim Dir(\gamma_{1},\gamma_{2},\ldots, \gamma_{K},\gamma_{u} )$ $\theta_{n} | \alpha, \pi\sim Dir(\alpha,\pi)$ $z_{jn} | \theta_{n} \sim Mult(\theta_{n})$ $e_{jn} | z_{jn}, \left( \beta_{k} \right)_{k=1}^{L} \sim F(\beta_{z_{jn}})$

$H \sim Dir(\eta)$ $\beta_{k} | H \sim H$

where $\gamma_{k}=\frac{\gamma}{L}, k=1..K$ and $\gamma_{u}=1-\sum_{k=1}^{K} \gamma_{k}$

and $F(\beta_{k})$ indicates the probability distribution of a CMN $k$ over all the possible edges in our vocabulary.

**Sampling**$\boldsymbol{z}_{\boldsymbol{jn}}$:

The terms $\theta$ can be integrated out because they are conjugate to the $z$ distribution. Thus, we can express $z$, given the mixture proportions, as:

$$p\left( z \right|\pi)= \prod_{n=1}^{N} \frac{\Gamma(\alpha)}{\Gamma(\alpha+j_{n})} \prod_{k=1}^{K} \frac{\Gamma(\alpha\pi_{k}+n_{nk})}{\Gamma(\alpha\pi_{k})}$$

Here, $n_{nk}$ is the number of edges in the network $n$ that belongs to the $k$-th mixture and $j_{n}$ is the total number of edges in the network $n$.

Then, an edge assignment $z_{jn}$ can be sampled, given the assignment of the other edges, as:

$$p\left( z_{jn}=k \right| z^{-jn}, \pi,e)= \left\{ \begin{aligned} \left( \alpha\pi_{k}+n_{nk}^{-jn} \right) f_{k}^{-e_{jn}}\left( e_{jn} \right) if k previously used \\ \\ \alpha\pi_{u} f_{k_{new}}^{-e_{jn}}\left( e_{jn} \right) otherwise \end{aligned} \right.$$

If a new mixture is created or no element is assigned to a previously existing mixture, the number of mixtures is adapted accordingly by increasing or decreasing the total number of mixtures K.

$f_{k}^{-e_{jn}}\left( e_{jn} \right)$ indicates the probability that an edge $e_{jn}$ to belong to the CMN $k$ given the information about the other edges. It is defined as follow :

$$f_{k}^{-e_{jn}}\left( e_{jn} \right)= \frac{\int f\left( e_{jn} \right|\beta_{k}) \prod_{j^{'},n^{'}\neq j,n, z_{jn}=k} f\left( e_{j^{'}n^{'}} \right|\beta_{k}) h\left( \beta_{k} \right) d\beta_{k}}{\int\prod_{j^{'},n^{'}\neq j,n, z_{jn}=k} f\left( e_{j^{'}n^{'}} \right|\beta_{k}) h\left( \beta_{k} \right) d\beta_{k}}$$

Where $h(.)$ is the density of $H$, which is in our case a $Dir(\eta)$. We replace every thing in the previous equation we get ( with a slight change of annotation to make it more readable)

$$f_{k}^{-e_{jn}}\left( e_{jn} \right)= \frac{\int\beta_{e_{jn}}^{\left( k \right)} \prod_{j^{'},n^{'}\neq j,n, z_{jn}=k} \beta_{e_{j^{'}n^{'}}}^{\left( k \right)} \frac{\Gamma\left( V\eta\right)}{\Gamma\left( \eta\right)^{V}} \prod_{v=1}^{V} \left( \beta_{v}^{\left( k \right)} \right)^{\eta-1} d\beta^{\left( k \right)}}{\int\prod_{j^{'},n^{'}\neq j,n, z_{ij}=k} \beta_{e_{j^{'}n^{'}}}^{\left( k \right)} \frac{\Gamma\left( V\eta\right)}{\Gamma\left( \eta\right)^{V}} \prod_{v=1}^{V} \left( \beta_{v}^{\left( k \right)} \right)^{\eta-1} d\beta^{\left( k \right)}}$$

$$= \frac{\int\beta_{e_{ij}}^{\left( k \right)} \prod_{v=1}^{V} \left( \beta_{v}^{\left( k \right)} \right)^{r_{v,k}^{-jn}}\prod_{v=1}^{V} \left( \beta_{v}^{\left( k \right)} \right)^{\eta-1} d\beta^{\left( k \right)}}{\int\prod_{v=1}^{V} \left( \beta_{v}^{\left( k \right)} \right)^{r_{v,k}^{-jn}} \prod_{v=1}^{V} \left( \beta_{v}^{\left( k \right)} \right)^{\eta-1}d\beta^{\left( k \right)}}$$

$$= \frac{\frac{\prod_{v=1}^{V} \Gamma(r_{v,k}^{-jn}+\eta+\delta\left( e_{jn},v \right) )}{\Gamma( \sum_{v=1}^{V} r_{v,k}^{-jn}+\eta+\delta\left( e_{jn},v \right))}}{\frac{\prod_{v=1}^{V} \Gamma(r_{v,k}^{-jn}+\eta)}{\Gamma( \sum_{v=1}^{V} r_{v,k}^{-jn}+\eta)}}$$

$$f_{k}^{-e_{jn}}(e_{jn})= \frac{r_{e_{jn},k}^{-jn}+\eta}{\sum_{v=1}^{V} r_{v,k}^{-jn}+\eta V}$$

And $f_{k_{new}}^{-e_{jn}}\left( e_{jn} \right)= \frac{1}{V}$ . Here, $r_{v,k}$ indicates the number of times the edge $v$ was assigned to CMN $k$.

**Sampling**$\boldsymbol{\pi}$:

In [[1](#_ENREF_1)], $\pi$ was sampled by introducing an auxiliary variable $m$ and expressing the second term in $p\left( z \right|\pi)$ as:

$$\prod_{k=1}^{K} \frac{\Gamma(\alpha\pi_{k}+n_{nk})}{\Gamma(\alpha\pi_{k})}=\sum_{m_{nk=0}}^{n_{nk}} s\left( n_{nk}, m_{jk} \right) \left( \alpha\pi_{k} \right)^{m_{nk}}$$

where $s\left( n, m \right)$ are Stirling numbers of the first kind such as:

$$s\left( n, m \right)= \left\{ \begin{aligned} 1 m=n=1 or 0 \\ 0 n>0, m=0 \\ 0 m>n \\ s\left( n+1, m \right)=s\left( n, m-1 \right)+ns\left( n,m \right) \end{aligned} \right.$$

Then, $m_{nk}$ can be sampled as follows:

$$p\left( m_{nk}=m \right|z, m^{-nk}, \pi)= \frac{\Gamma(\alpha\pi_{k})}{\Gamma(\alpha\pi_{k}+n_{nk})} s\left( n_{nk}, m \right)\left( \alpha\pi_{k} \right)^{m}$$

Then, we can sample $\pi$ as:

$$\pi\sim Dir(\sum_{n=1}^{N} m_{n1}, \ldots., \sum_{n=1}^{N} m_{nK}, \gamma)$$

In our case the first and second level concentration parameters $\gamma$ and $\alpha$ where set to 1. The parameter $\eta$ was set to 0.01 (to allow for sparsity).

1. Yee Whye T, Michael IJ, Matthew JB, David MB: **Hierarchical Dirichlet Processes.** *Journal of the American Statistical Association* 2006, **101**.
