# Supplementary material for: 3CPET: finding co-factor complexes from ChIA-PET data using a hierarchical Dirichlet process
Source: Genome Biol. 2015 Dec 22;16:288. doi: 10.1186/s13059-015-0851-6 (PMC4716632; doi:10.1186/s13059-015-0851-6)
Supplement: Additional file 3: — All supplementary figures (S1 to S23) and their corresponding legends. (DOCX 6465 kb) [file 13059_2015_851_MOESM3_ESM.docx]

# Supplementary Figures for :

# 3CPET: Finding Co-factor Complexes from ChIA-PET data by using Hierarchical Dirichlet Process

**Mohamed Nadhir Djekidel1,** **Zhengyu Liang^1^, Qi Wang^1^, Zhirui Hu^1^, Guipeng Li^1^, Yang Chen^1^**^§^**, Michael Q. Zhang^1,2^** ^§^

^1^ MOE Key Laboratory of Bioinformatics and Bioinformatics Div, Center for Synthetic and System Biology, TNLIST /Department of Automation, Tsinghua University, Beijing 100084, China;

^2^ Department of Molecular and Cell Biology, Center for Systems Biology, The University of Texas, Dallas 800 West Campbell Road, RL11 Richardson, TX 75080-3021, USA


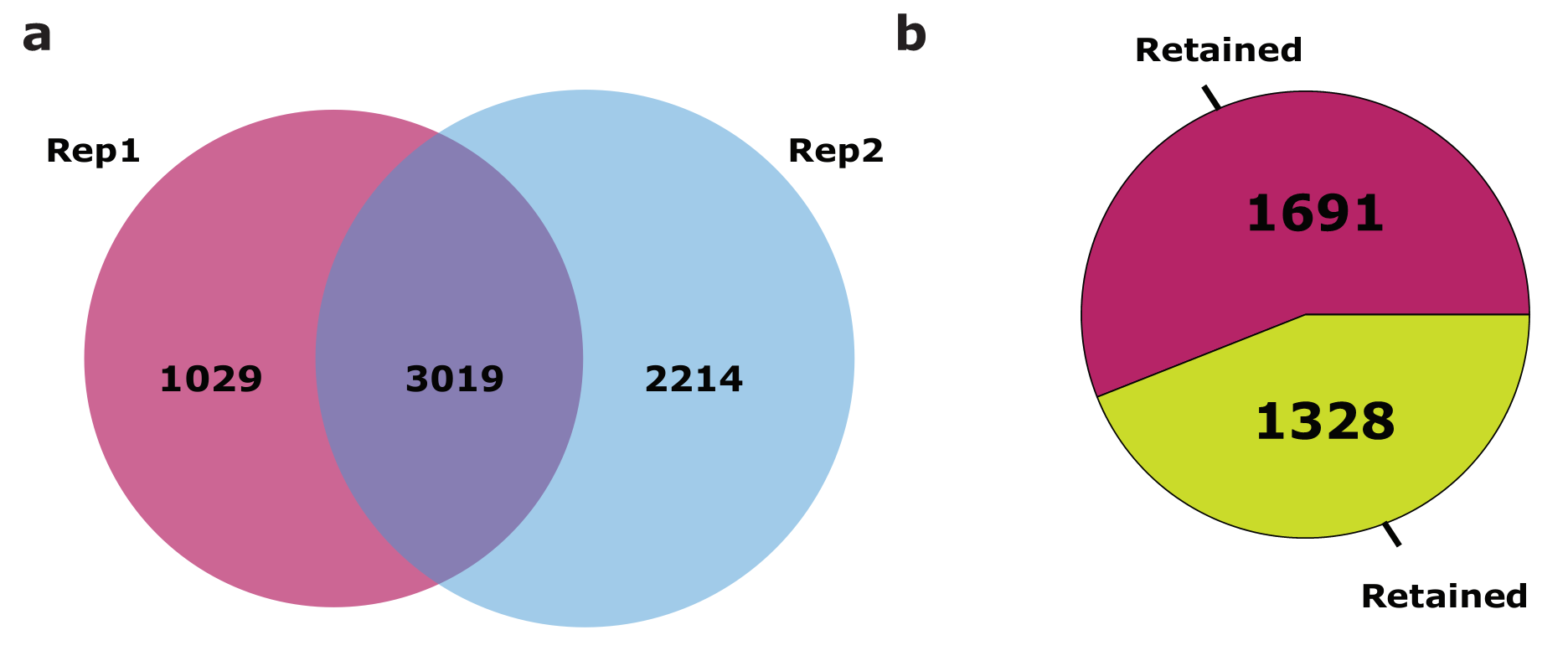


**Supplementary figure S1 | MCF7 Raw data statistics. a)** *Common interactions between ER-alpha ChIA-PET replicates in MCF7 cell-data (GSM970212).* **b)** *From the 3019 interactions we only considered 1691 interactions that have a frequency of three or more.*


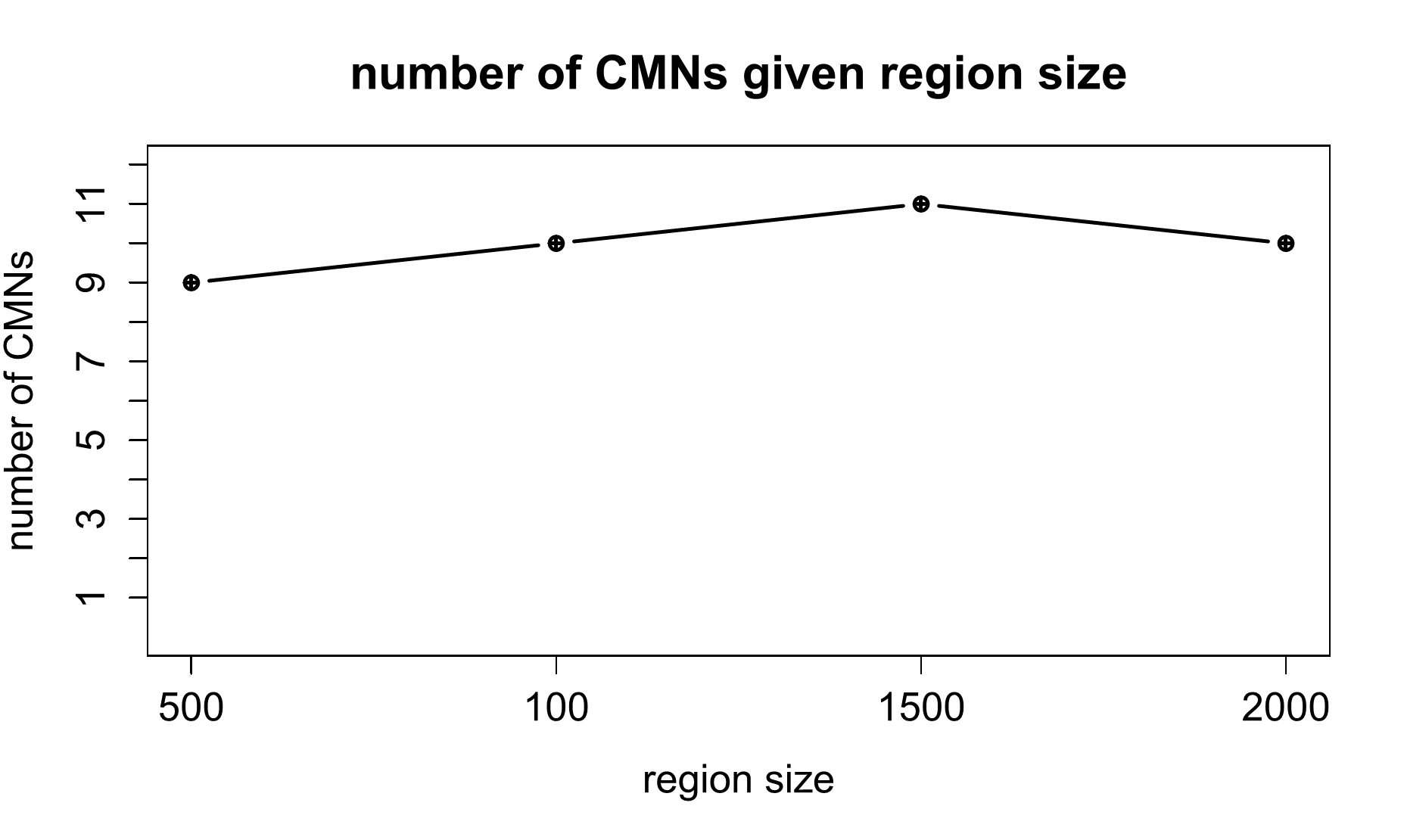


**Supplementary figure S2 | Number of inferred ER-alpha CMNs per region size*.*** *3CPET results using different region size around the centers of PETs. We can see that overall about a number of 10 networks is inferred.*


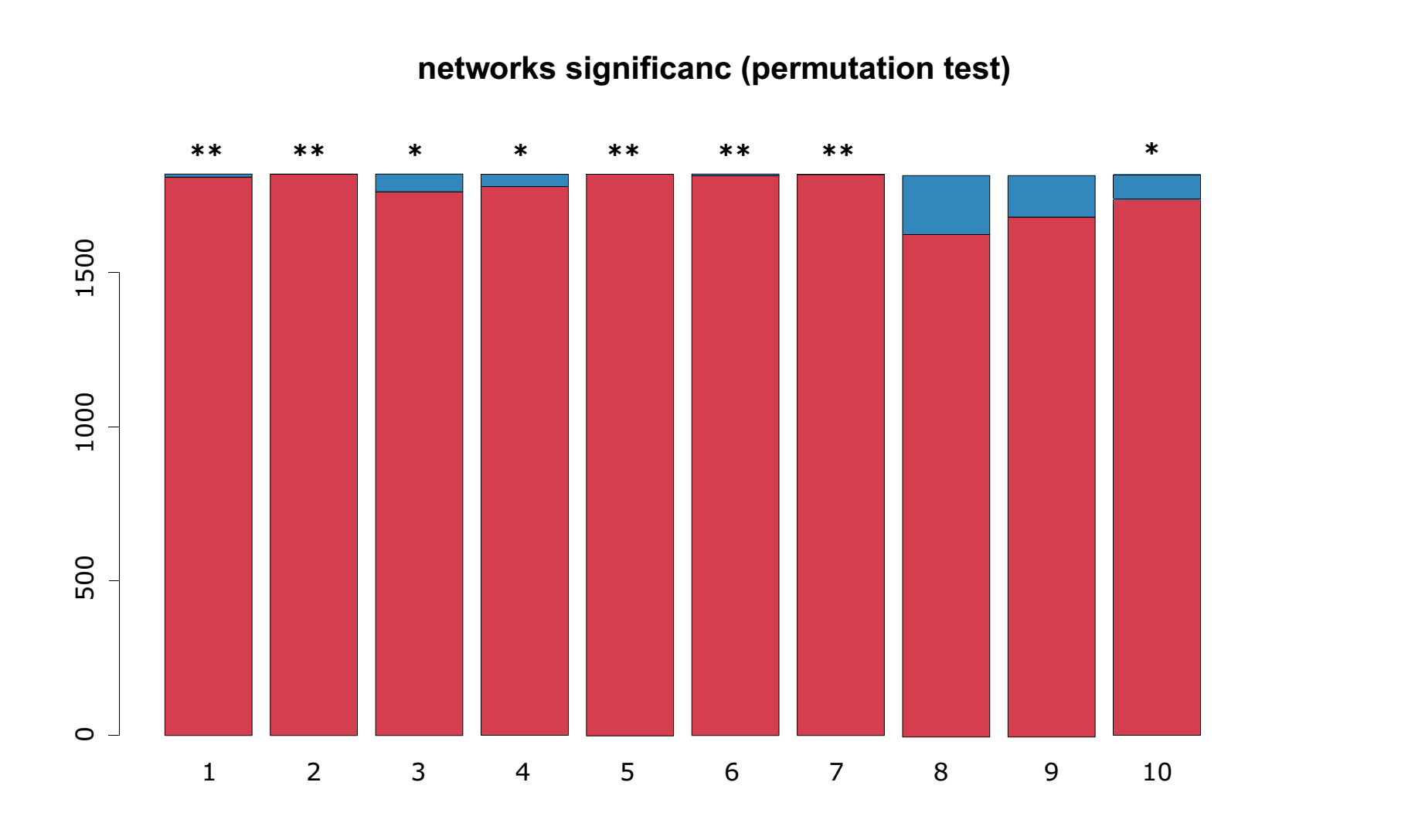


**Supplementary figure S3 | ER-alpha inferred CMNs significance test*.*** *A permutation test was performed in which we permuted the wiring of the networks by permuting the position of the TF between the DNA regions, then, we checked the probability of obtaining one of the inferred networks by chance. The red color represents the number of trials where our results were significantly non-random. The blue color indicated the number of times the network was similar to a random network.*


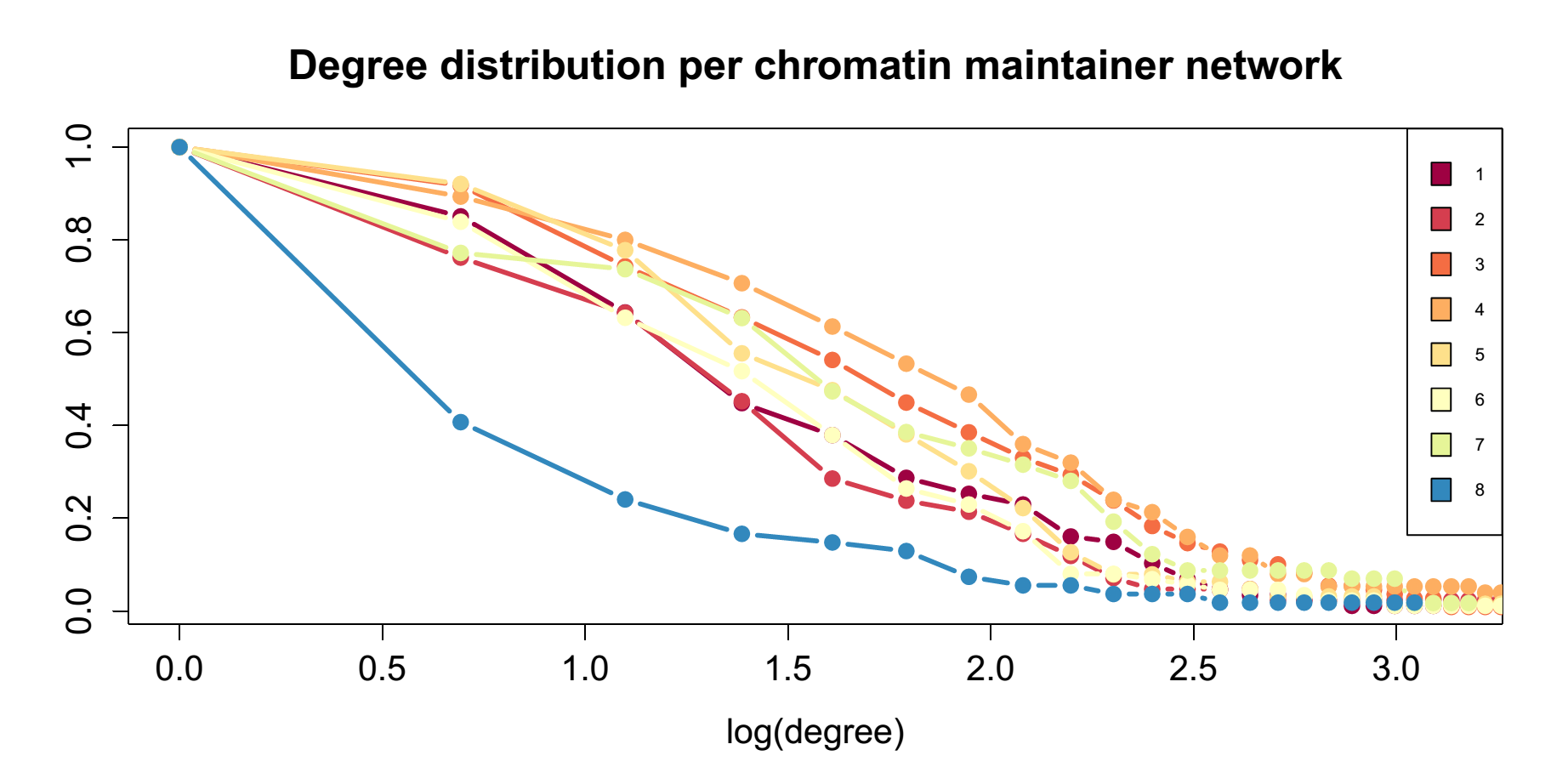


**Supplementary figure S4 | Degree distribution of ER-alpha inferred CMNs.** *The inferred networks exhibit a small-world network structure with a small number of hub proteins playing the role of backbone of the network and the other proteins with small number of interactions.*


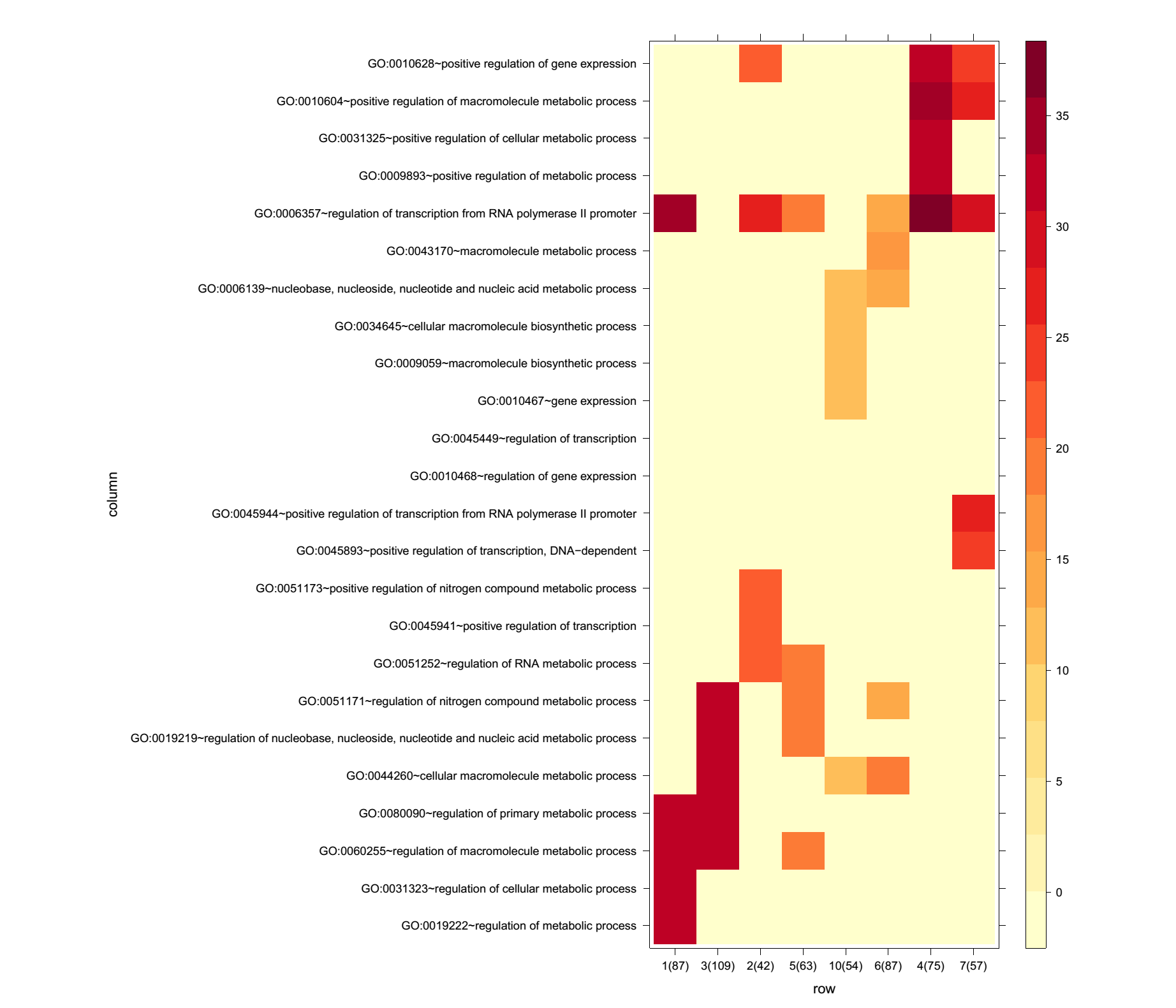


**Supplementary figure S5 | GO-based function enrichment of the proteins composing the different CMNs.** *Almost all the CMNs are composed with proteins involved in transcription regulation (GO:0006357) except CMN 3, which is composed of elements involved in regulating metabolic process, and CMN10, however, CMN 10 shows a significant enrichment gene expression regulatory proteins.*


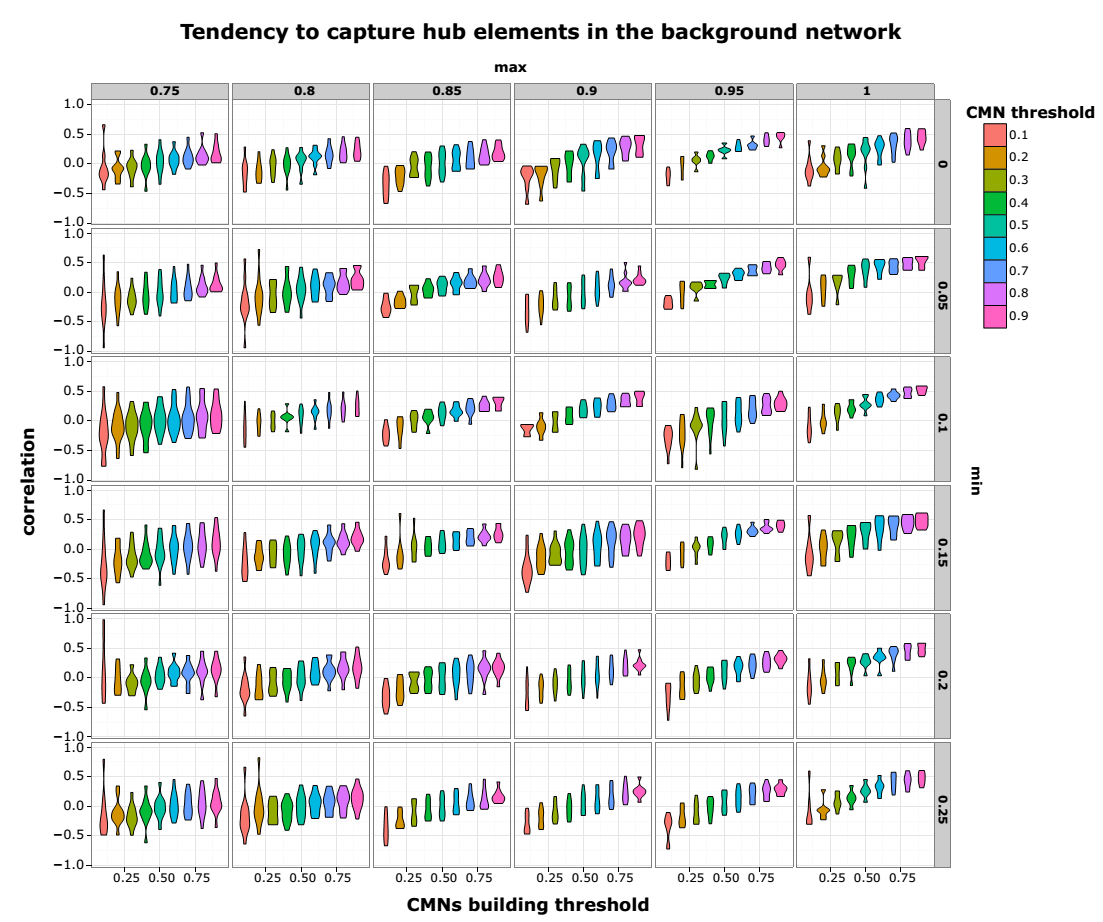


**Supplementary figure S6 | Influence of the background PPI structure on the results.** *Each tile represent the relation between the CMNs building threshold and correlation between the degree of the predicted proteins in the CMNs and the background PPI organized by the min and max filtering thresholds. The plot indicates that hub proteins in background PPI tend to be captured by 3CPET, which indicates that the background PPI construction and filtering step is very crucial.*


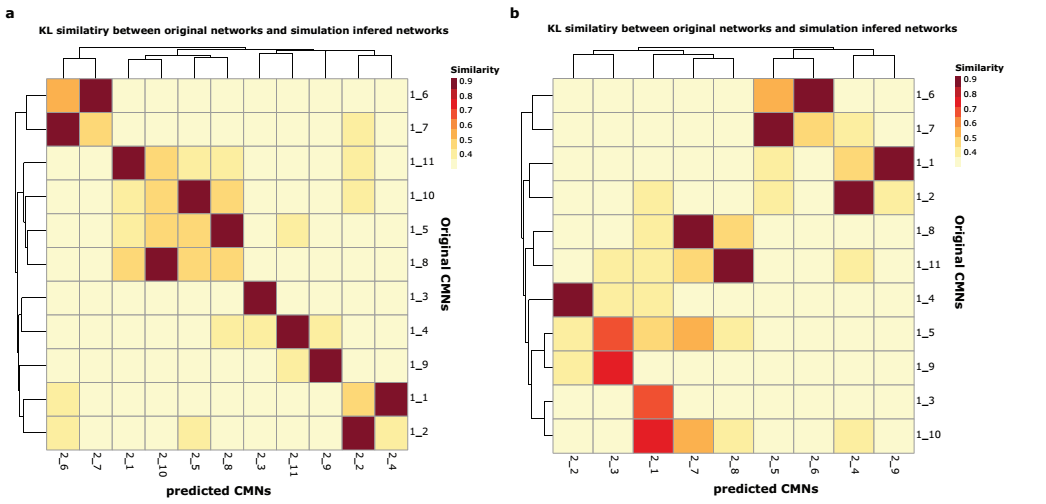


**Supplementary figure S7 | Example showing the similarity of predicted and original CMNs in simulation results. a)** *Heatmap showing the KL similarity between the predicted CMNs and the original CMNs in the simulated data, in this case 3CPET was able to predict all the CMNs.* ***b)*** *heatmap showing a case in which 3CPET predicts less CMNs, we notice that some the predicted CMNs are a mixture of original CMNs.*


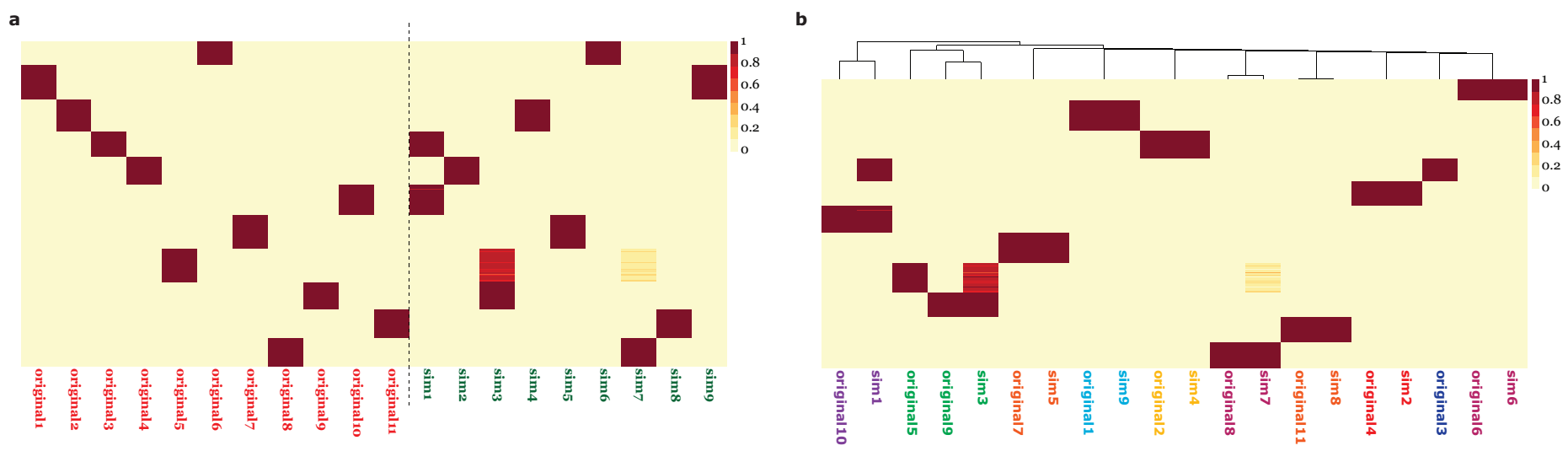


**Supplementary figure S8 | Example showing the recovery of the enrichment of interactions in the simulation results. a)** shows a case in which 3CPET predicts less CMNs than the actually existing ones. *Left part (in red labels) show the simulated enrichment of the different CMNs in the different Chromatin interactions. The right part (green labels) shows the predicted enrichment of the CMNs predicted by 3CPET.* ***b)*** *Clustering of the profiles in figure a. We notice that the predicted CMNs are enriched in the same regions are the original one. As the predicted number is less than the original, some CMNs are enriched in a mixture of two original CMNs.*


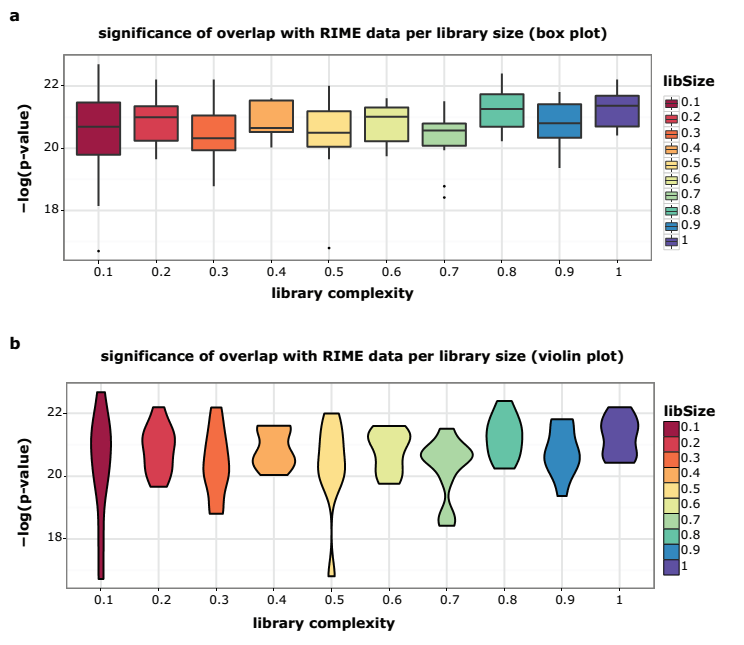


**Supplementary figure S9 | Significance of the overlap of 3CPET results with RIME data per library complexity.** *Library complexity indicates the percentage of significant interactions detected from the total data. The plots shows the distribution p-values obtained by checking the overlap of 3CPET results with RIME data per library size.* ***a)*** *For each library complexity x, we generated 10 samples of size x% sampled from the original ChIA-PET data. The box-plots indicate that the mode of the overlap p-value shows an increasing trend .* ***b)*** *Violin plot of the same data in figure a, just to make the variability of the results more visible. We notice that the overlap p-value becomes more stable with increasing experiment quality.*


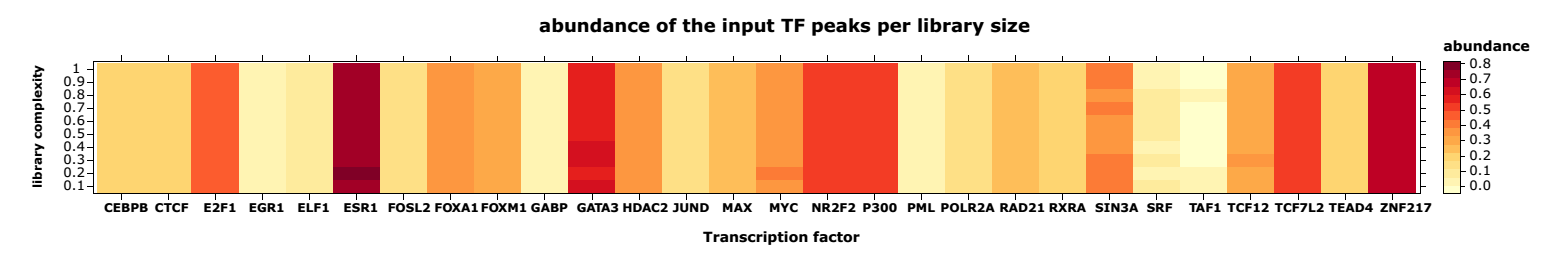


**Supplementary figure S10 | Abundance of the TF Chip-Seq signal per library size.** *We notice that the strongly associated TF with ER-alpha are highly enriched in ChIA-PET interactions regardless of the library complexity. This can explain the more or less stable overlap p-values with RIME data starting from a 0.2 library size.*


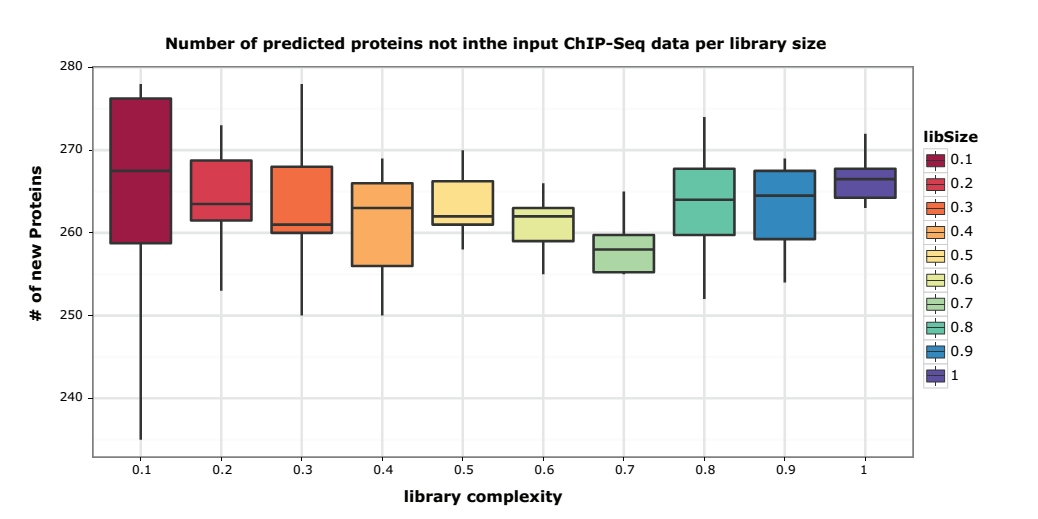


**Supplementary figure S11 | Abundance of the TF Chip-Seq signal per library size.** *We notice that the strongly associated TF with ER-alpha are highly enriched in ChIA-PET interactions regardless of the library complexity. This can explain the more or less stable overlap p-values with RIME data starting from a 0.2 library size.*


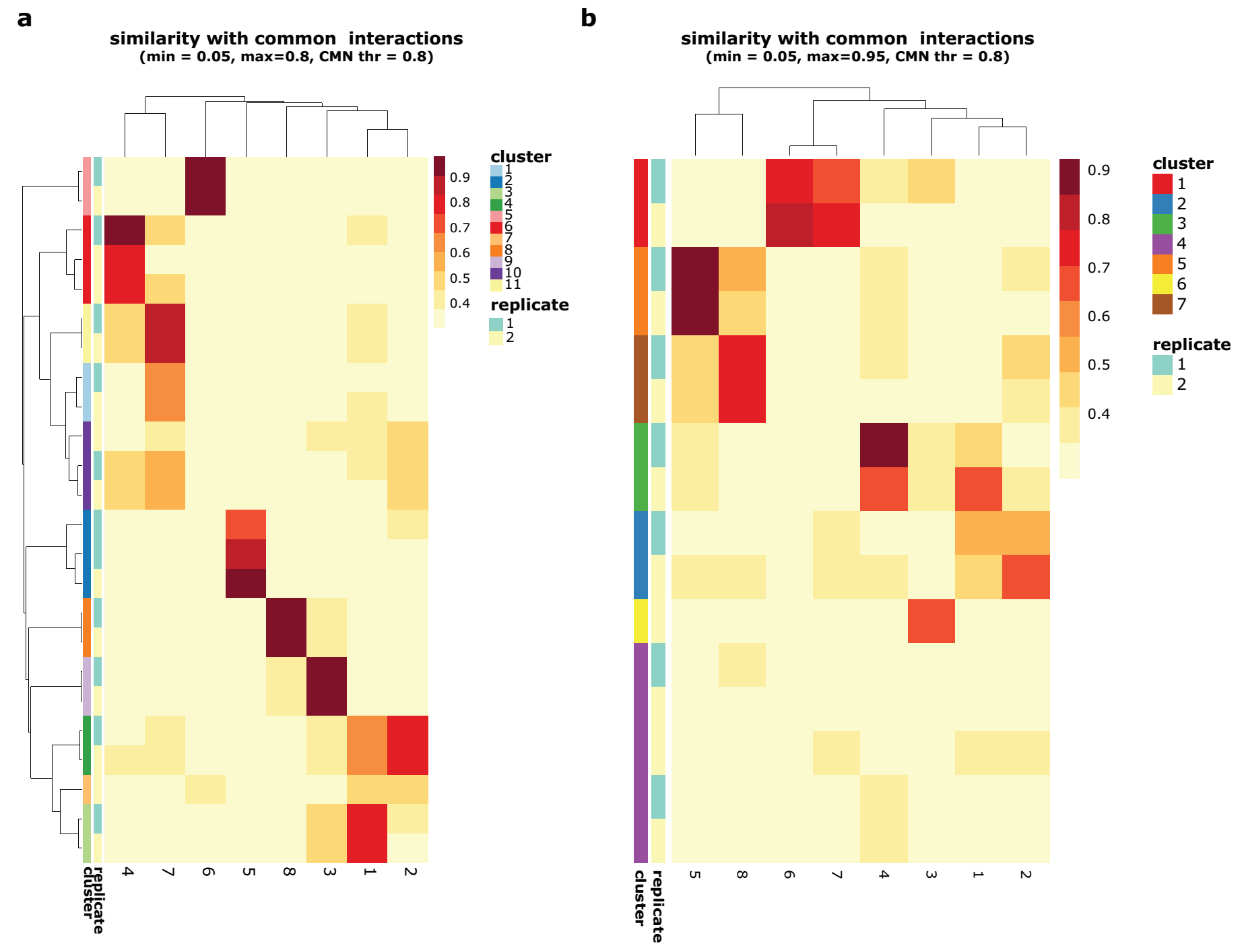


**Supplementary figure S12 | Robustness analysis.** *shows the clustering of the CMNs obtained using the ER-alpha ChIA-PET replicates according to their similarity to the CMNs obtained using common interactions using different thresholds.* ***a)*** *Using a min/max threshold of 0.05 and 0.8 respectively leads to more robust predictions, we notice that except the CMN of replicate 2 in cluster 8 (orange color), all the other predicted CMNs show a similarity to the common interactions CMNs.* ***b)*** *loosing a bit the filtering threshold lead to the introduction of new proteins and leads to the prediction of new CMNs not similar to the ones obtained using common chromatin interactions (cluster 4, purple color)*

**

**Supplementary figure S13| Example showing the behavior of Dirichlet distribution in a 3-dimensional simplex.** *Here we plot the pdf of the Dirichlet distribution on a 3-dimensional simplex. Here we consider each point to be a CMN or an edge. We notice that* $\eta$ *values less than 1 puts more weight on each edge in figures* ***a)*** *and* ***b).*** *A value of 1 gives a uniform distribution* ***c).*** *In* ***d)*** *and* ***e)*** *we can see that* $\eta$ *larger than 1 concentrate the weight on the center of the simplex given equal probability to observer the different edges.*

*l*

**Supplementary figure S14| Influence of HDP parameters on edge-to-CMNs sparsity .** *Each tile plot shows the effect of varying* $\eta$ *for a fixed* $\gamma$ *and* $\alpha$ *values. The sparsity here is calculated by counting the number of non-assigned CMNs for each edge. We notice that regardless of* $\gamma$ *and* $\alpha$*, increasing* $\eta$ *reduces the level of sparsity, hence, enabling an edge to be part of many CMNs. We also notice that the level of sparsity changes when* $\gamma\leq1$ *and* $\gamma>1$*. With larger* $\gamma$ *values inducing more sparcity for a fixed* $\eta$ *and* $\alpha$*.*

**

**Supplementary figure S15| Influence of HDP parameters on PPI-to-CMNs sparsity .** *Each tile plot shows the effect of varying* $\eta$ *for a fixed* $\gamma$ *and* $\alpha$ *values. Here we calculated the distribution of the number local PPI controlled by each CMN. Smaller number indicates a sparse PPI-to-CMN matrix, while larger values indicate less sparsity. We notice that generally, increasing* $\eta$ *reduces the level of sparsity, hence, enabling an edge to be part of many CMNs.*

**

**Supplementary figure S16| Influence of HDP parameters on the number of inferred CMNs .** *Each tile plot shows the effect of varying* $\eta$ *for a fixed* $\gamma$ *and* $\alpha$ *values. Here we calculated the distribution of the number of inferred CMNs in each setting. We notice that generally, increasing* $\eta$ *leads to less inferred CMNs. We also notice that* $\gamma$ *values larger than 1 leads to the inference of more CMNs.*


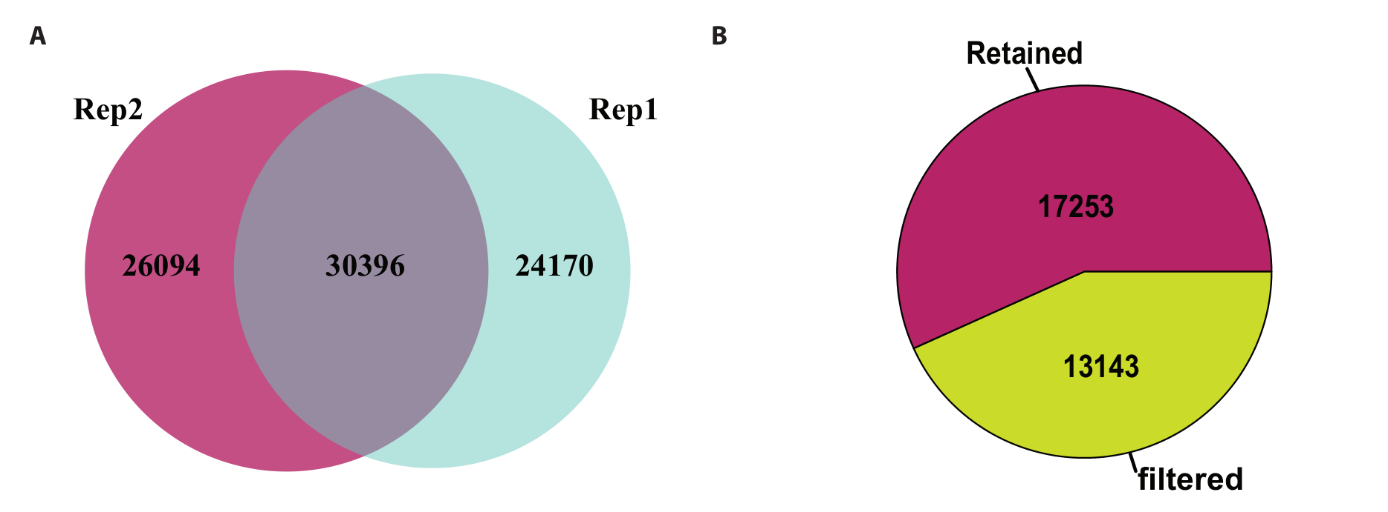


**Supplementary figure S17 | K562 Raw data statistics. *a)*** *Venn diagram of the number of common interactions between K562 RNAP-II ChIA-PET replicates.* ***b)*** *Common interactions A) where filtered by considering only interactions with frequency > 5 , about 17253 interactions were retained.*


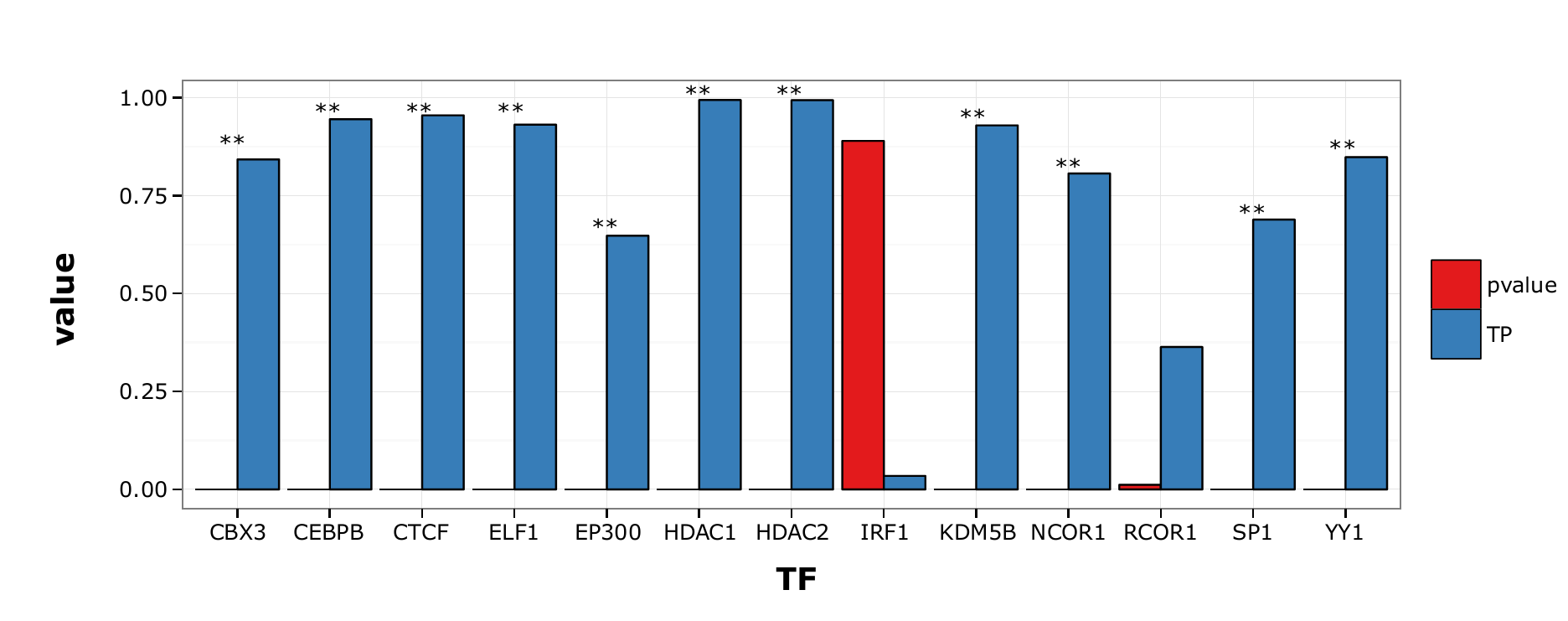


**Supplementary figure S18 | ER-alpha inferred CMNs significance test.**


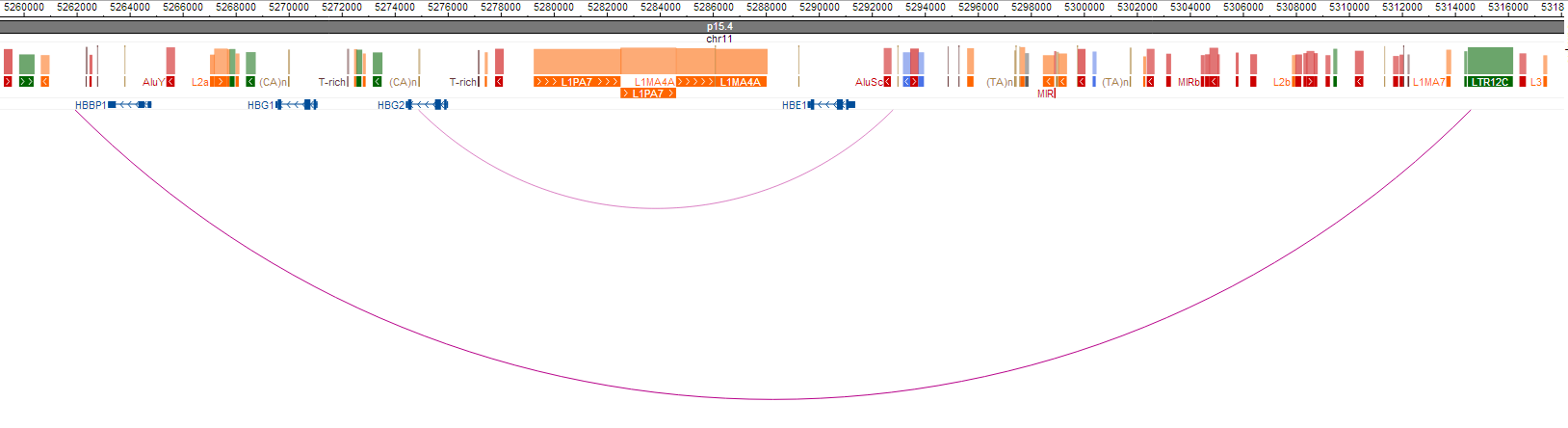


**Supplementary figure S19 |** $\boldsymbol{\beta}$**-globin locus used loops.** *Two* $\beta$*-globin loops were used by 3CPET while the others were filtered because they had an interaction frequency less than 5 or 3CPET was not able to construct a network connecting its both DNA ends. The outer-loop was predicted to be maintained by CTCF and GAT1 while the inner-loop involved transcription related proteins.*


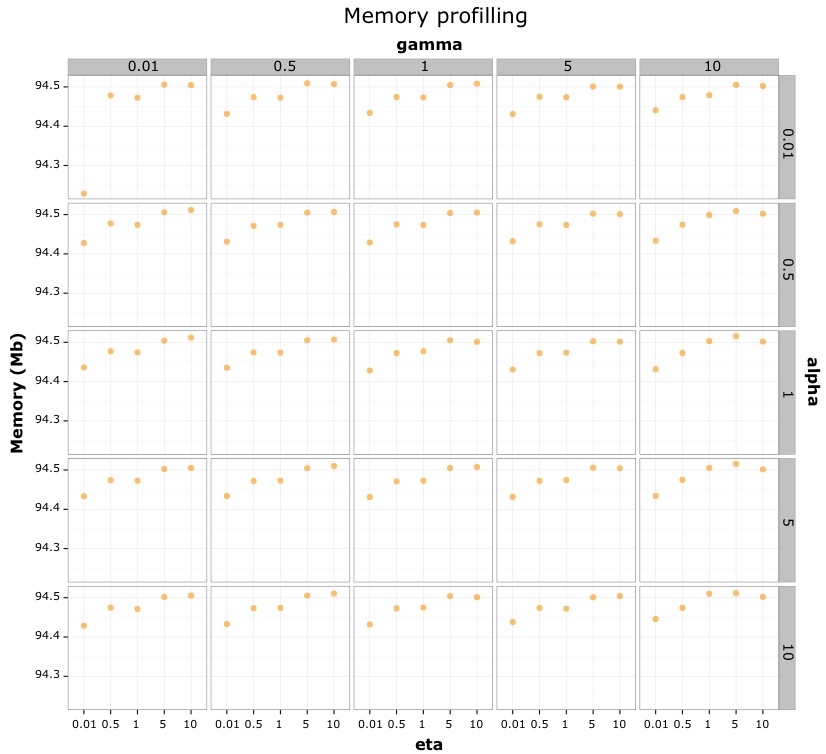


**Supplementary figure S20 | Memory occupancy of 3CPET given different HDP parameters.** This tile plot shows the memory occupancy of R3CPET given HDP parameters given ER-alpha interactions. We notice that for more than 1600 interactions, 3CPET generally occupies from 94Mb~100Mb memory which is reasonable on actual machines. The increase of $\eta$ values lead to an increase of 1Mb ~ 2 Mb, as less sparcity is introduced.


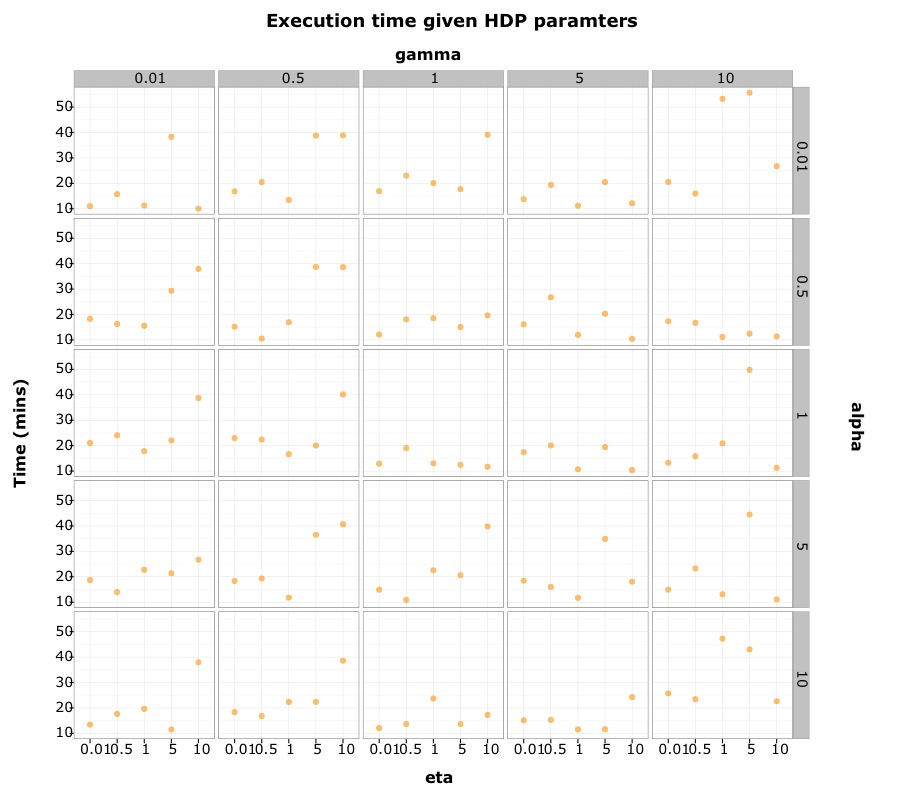


**Supplementary figure S21 | Running time of 3CPET given different HDP parameters.** This tile plot shows the running time of R3CPET on ER-alpha interactions. We notice that there is not a fixed pattern as the results depend on Gibbs sampling, but we observe that large $\eta$ values tend to lead 3CPET to run longer than when using smaller values as we less sparsity is introduced which increases calculations.

**Supplementary figure S22 | Running time of 3CPET given the number of inferred CMNs.** In this plot we plotted the relation between the number of CMNs and the execution time. We notice that generally the number of inferred CMNs does not have a big influence on the running time and it is generally local

**Supplementary figure S23 | Running time and memory occupancy given the input data size.** These plot show the time and memory scaling of 3CPET given increasing the number of DNA-DNA interactions provided as input. Here the time is shown for $\eta=0.01, \gamma= \alpha=1$. Similar trend can be seen in the other settings. We notice that the time and memory requirements of 3CPET scales linearly with increasing data size.


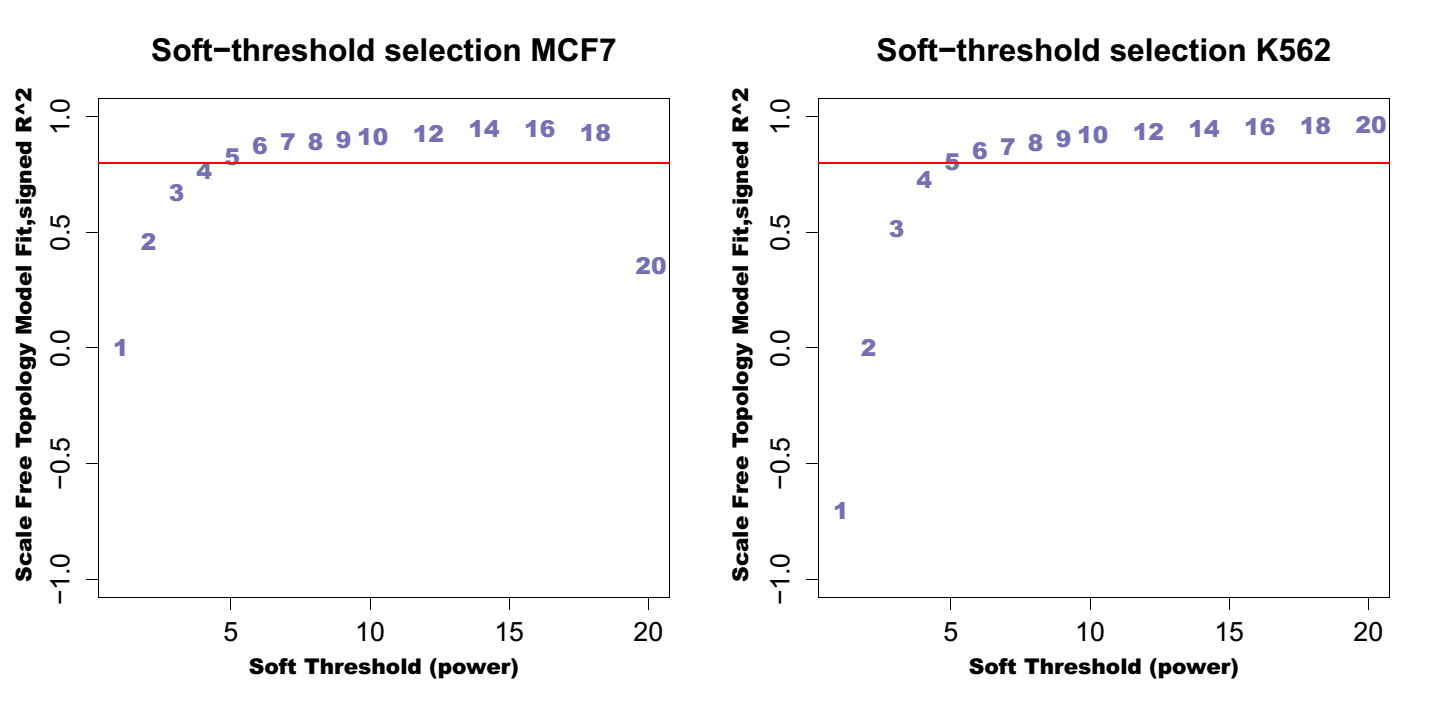


**Supplementary figure S23 | Soft-threshold selection for weighting the edges MCF7 and K562 co-expression networks.** In order to build a co-expression network, gene interactions should weighted. In the WCGNA, a soft thresholding power $\beta$ should be selected to approximate scale-free topology. We selected a $\beta$ that have an $R^{2}$ score of at least 0.8 when fitting a scale-free topology.
