# Supplementary material for: 3CPET: finding co-factor complexes from ChIA-PET data using a hierarchical Dirichlet process
Source: Genome Biol. 2015 Dec 22;16:288. doi: 10.1186/s13059-015-0851-6 (PMC4716632; doi:10.1186/s13059-015-0851-6)
Supplement: Additional file 4: — All supplementary tables (S1 to S3) and their corresponding legends. (DOCX 15 kb) [file 13059_2015_851_MOESM4_ESM.docx]

| **TF** | **Total number of peaks** | **Number of interacting regions** |
| --- | --- | --- |
| CEBPB | 13302 | 657 |
| CTCF | 58994 | 663 |
| E2F1 | 21946 | 1545 |
| EGR1 | 1735 | 70 |
| ELF1 | 8161 | 339 |
| ESR1 | 9112 | 2539 |
| FOSL2 | 5199 | 468 |
| FOXA1 | 12661 | 1157 |
| FOXM1 | 1841 | 965 |
| GABP | 5834 | 132 |
| GATA3 | 20950 | 2013 |
| HDAC2 | 6687 | 1228 |
| JUND | 2389 | 489 |
| MAX | 11661 | 861 |
| MYC | 24587 | 1292 |
| NR2F2 | 11361 | 1800 |
| P300 | 3346 | 1818 |
| PML | 4538 | 190 |
| POLR2A | 16136 | 480 |
| RAD21 | 34228 | 878 |
| RXRA | 3698 | 661 |
| SIN3A | 8791 | 1306 |
| SRF | 5050 | 210 |
| TAF1 | 1864 | 28 |
| TCF12 | 2215 | 1102 |
| TCF7L2 | 17899 | 1758 |
| TEAD4 | 2133 | 658 |
| ZNF217 | 19211 | 2232 |

**Supplementary table S1 |Chip-Seq signals as input in the ER-alpha ChIA-PET case.** Statistics about the number of interacting region calculated used 1.5kb region size.

| **TF** | **Total number of peaks** | **Number of interacting regions** |
| --- | --- | --- |
| ATF3 | 1918 | 1999 |
| BDP1 | 1409 | 739 |
| BRF1 | 330 | 394 |
| BRF2 | 221 | 245 |
| EGR1 | 2946 | 4757 |
| FOS | 20249 | 11086 |
| GABPA | 3648 | 5660 |
| GATA1 | 5492 | 3801 |
| GATA2 | 12483 | 7241 |
| GTF2B | 4626 | 10867 |
| GTF3C2 | 5870 | 6355 |
| HEY1 | 28945 | 39016 |
| JUN | 56008 | 30501 |
| JUND | 1499 | 1592 |
| MAX | 10475 | 12259 |
| MYC | 27322 | 26625 |
| NFE2 | 5069 | 2329 |
| NFYA | 13048 | 7923 |
| NFYB | 15608 | 8990 |
| POLR2A | 76821 | 6542 |
| POLR3A | 2708 | 1639 |
| RAD21 | 28615 | 5699 |
| RDBP | 1739 | 5103 |
| REST | 2739 | 565 |
| SIN3A | 1472 | 3858 |
| SIRT6 | 6527 | 4119 |
| SIX5 | 3419 | 5534 |
| SMARCA4 | 14704 | 9732 |
| SMARCB1 | 16003 | 9521 |
| SPI1 | 46201 | 20312 |
| SRF | 668 | 791 |
| STAT1 | 8875 | 10184 |
| STAT2 | 8105 | 9951 |
| TAF1 | 11844 | 21951 |
| USF1 | 9555 | 7167 |
| XRCC4 | 349 | 730 |
| ZNF263 | 10057 | 10527 |

**Supplementary table S2 |Chip-Seq signals as input in the Pol-II ChIA-PET case.** The statistics about the number of interacting regions calculated used 1.5kb region size.

|  | $V_{1}$ | $V_{2}$ | $V_{3}$ | $V_{4}$ | $V_{5}$ | $V_{6}$ | $V_{7}$ | $V_{8}$ |
| --- | --- | --- | --- | --- | --- | --- | --- | --- |
| $\beta$-Globin | 1.7e-03 | 8.8e-03 | 8.8e-03 | 0.23 | 8.9e-04 | 8.8e-03 | 8.9e-04 | 5.4e-03 |
| transcription | 7.2e-03 | 0.587 | 7.2e-03 | 0.866 | 0.026 | 0.026 | 7.2e-03 | 7.2e-03 |

**Supplementary table S3 |Literature mining results.** Enrichment p-values for one network compared to all the predicted proteins for the beta-globin and transcription related literature mining networks.
