# Supplementary material for: 3CPET: finding co-factor complexes from ChIA-PET data using a hierarchical Dirichlet process
Source: Genome Biol. 2015 Dec 22;16:288. doi: 10.1186/s13059-015-0851-6 (PMC4716632; doi:10.1186/s13059-015-0851-6)
Supplement: Additional file 5: — 3CPET package manual. (PDF 976 kb) [file 13059_2015_851_MOESM5_ESM.pdf]

R3CPET user manual

Mohamed Nadhir Djekidel, Yang Chen et al
nde12@mails.tsinghua.edu.cn

August 8, 2015

Contents

1 Introduction **2**

2 Using *R3CPET* **3**
2.1 Loading data 4
2.2 Creating indexes 6
2.3 building networks for DNA regions 6
2.4 Inferring chromatin maintainer networks 7
2.5 Cluster DNA interactions by enrichment profile 9
2.6 Visualization 9
2.6.1 Heatmaps 10
2.6.2 Enrichment curves 11
2.6.3 clusters pair-wise scatter plot 12
2.6.4 plot networks 13
2.6.5 Networks similarity 14
2.6.6 Circos maps 15
2.7 Gene enrichment 16
2.7.1 Networks GO enrichment 16
2.7.2 GO enrichment of the genes in each cluster 16
2.8 Using the web interface 17
2.8.1 Raw data visualization 18
2.8.2 Results visualization 18

3 Session Info **19**

1 Introduction

The breakthrough in chromatin conformation study done in the last decade has shed some light on many aspects of gene transcription mechanisms and revealed that chromatin crosstalk plays an important role in connecting regulatory sequences to their target promoters through loop formation

However, a key limitation of the existing chromatin conformation assays is that they can just give us the genomic coordinates of the interacting DNA fragments but don't tell us on the proteins involved.One used solution is to use *Chip-Seq* to get the list of the proteins involved at these borders but one limitation is that not all the proteins can be captured and some don't have a specific anti-body.

Thus, computational methods are still useful to give some insight on the protein candidates that can play a role in maintaining these interactions. *R3CPET* comes as a tool to fill this gap and try to infer the loop-maintaining network(s) in a more concise manner.

3CPET is based on the following idea : if we can have the list of the protein networks that maintain all of the DNA-interactions, then we can infer the set the most enriched networks. One of the widely used statistical model in this kind of problems is the HLDA model which is a non-parametric Bayesian that enables us to infer the number of different groups from the data.

In order to apply HLDA, we need to have a corpus of documents and we assign each word to a topic. In our case each document is a network and each word is an edge in that network. To create a network for each interaction we use *(i)* find the set of the proteins involved a the boundaries of a loop and this by using *Chip-Seq* peaks or *motifs* data.

In the next step, we use the information in a background PPI to construct the network connecting the two interacting DNA fragments.

The networks are then converted into a *bag* of edges (*i.e* allow repetition) and fed to the HLDA algorithm.

In this model, we suppose that each network $(j_n)_{n=1}^{\infty}$ is made-up of a mixture of protein complexes each with different a proportion $\theta_n \sim DP(\alpha, \pi)$. To infer the number of clusters, the model suppose that we have an infinite

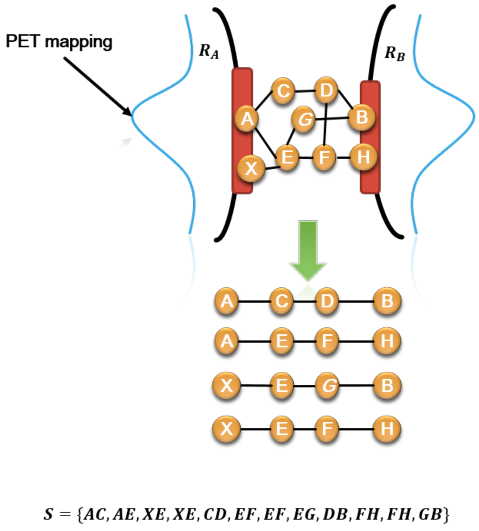

Figure 1: Illustration of the network construction procedure.

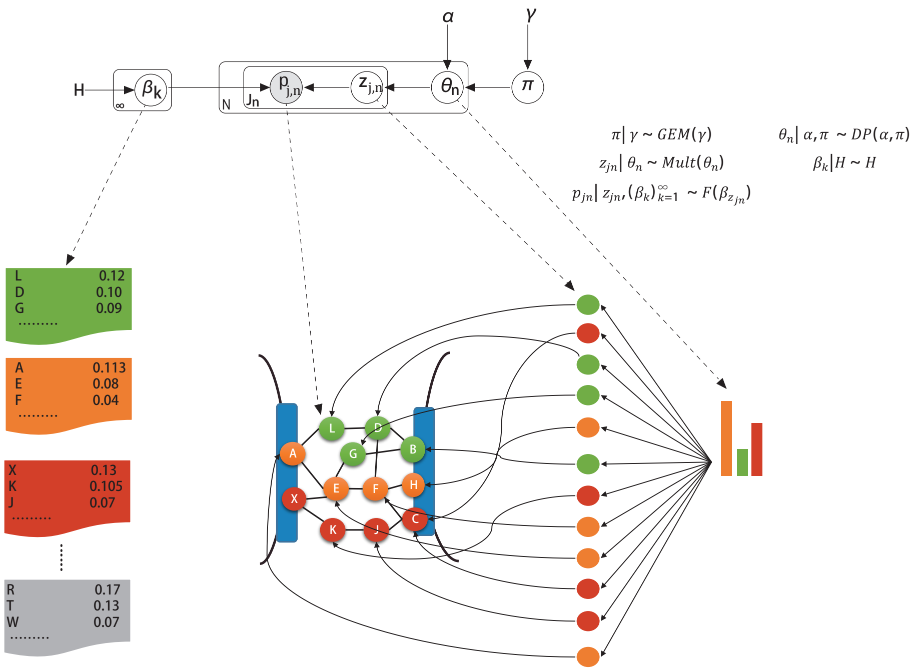

Figure 2: HLDA model

number of chromatin maintainer networks distributed according to a certain distribution H. To enable the sharing the complexes (β_k) across all the networks, an intermediate discrete distribution is introduced $(\beta_k)_{k=1}^{N}$ sampled from the base distribution H using a stick breaking construction $\pi|\gamma \sim GEM(\gamma)$.

2 Using *R3CPET*

R3CPET package were built to enable the user to upload the data step-by-step and thus giving him a more granular control of the data that he uses. Maybe it sounds like the use needs more labor, but the user can create an `S3` wrapper function that encapsulate the workflow functions into one method.

Four main classes are provided by *R3CPET*:

1. `ChiapetExperimentData` - a container for the raw data to use. 3 types of data can be loaded into this class respectively :*ChIA-PET* interactions, *ChIP-Seq* peaks and the background *PPI*.
2. `NetworkCollection` - Holds the list of the build networks for each DNA-interaction and their information.
3. `HLDAResult` - contains the results of the HLDA algorithm.
4. `ChromMaintainers` - contains the final results after processing the HLDA results (*i.e*: list of networks and their nodes).

In addition to these classes additional helper methods for GO enrichment and and gene conversion.

2.1 Loading data

Before starting the analysis different kinds of dataset need be loaded. This can be done using a `ChiapetExperimentData` object.

The user can load the data using the `ChiapetExperimentData` constructor. By passing: ChIA-PET interactions data, transcription factors binding site (TFBS) and a protein-protein interaction network. The ChIA-PET interactions can be passed as path to a file or a `GRanges` object. The same is for the TFBS.

```
> library(R3CPET)
> petFile <- file.path(system.file("example", package = "R3CPET"),
+     "HepG2_interactions.txt")
> tfbsFile <- file.path(system.file("example", package = "R3CPET"),
+     "HepG2_TF.txt.gz")
> x <- ChiapetExperimentData(pet = petFile, tfbs = tfbsFile, IsBed = FALSE,
+     ppiType = "HPRD", filter = TRUE)
```

Three types of data can be loaded :

- *ChIA-PET interactions* - which can have two formats: (i) the first type is a file in which the first six columns indicate the left and right interacting parts (generally parsed from the ChIA-PET tool). For example

```
> petPath <- system.file("example", "HepG2_interactions.txt", package = "R3CPET")
> petFile <- read.table(petPath, sep = "\t", header = TRUE)
> head(petFile)
##   chromleft startleft endleft chromright startright
## 1      chr1   1241413 1242055       chr1    1242973
## 2      chr1   1282738 1283678       chr1    1283743
## 3      chr1   1370817 1371926       chr1    1372332
## 4      chr1   1370817 1371926       chr1    1373404
## 5      chr1   1406249 1407391       chr1    1408030
## 6      chr1   1508839 1510117       chr1    1510147
##   endright counts       pvalue  qvalue
## 1  1243475      4 3.195947e-16 8.8e-05
## 2  1284926      6 1.075586e-21 8.8e-05
## 3  1373312      8 3.536936e-26 8.8e-05
## 4  1373766      4 7.962512e-14 8.8e-05
## 5  1408497      3 4.378941e-13 8.8e-05
## 6  1511194      4 2.172431e-14 8.8e-05
```

  Here only the 6 first columns are considered. It is up to the user to filter the significant interactions for him. The second type of files that can be loaded is a four columns file in which the first three columns indicate the genomic location of a DNA region and the forth column indicate if the region is located at the right or left side. The IDs in the fourth column should have the pattern `PET#\\d+\\.1` for the left side and `PET#\\d+\\.2` for the right side. if the number of the left side interactions is different from the right side an error will be raised.

```
> petPath <- system.file("example", "HepG2_centered.bed", package = "R3CPET")
> petFile <- read.table(petPath, sep = "\t", header = FALSE, comment.char = "+")
>
> head(petFile)
##     V1      V2      V3      V4
## 1 chr1 1241234 1242234 PET#1.1
## 2 chr1 1242724 1243724 PET#1.2
## 3 chr1 1282708 1283708 PET#2.1
## 4 chr1 1283834 1284834 PET#2.2
```

```
## 5 chr1 1370871 1371871 PET#3.1
## 6 chr1 1372322 1373322 PET#3.2
```

The method `loadPETs` can be used to load the data

```
> ## if it has 6 columns format IsBed = FALSE
> petPath <- system.file("example", "HepG2_interactions.txt", package = "R3CPET")
> x <- loadPETs(x, petFile = petPath, IsBed = FALSE)
## 870 interacting DNA regions loaded
```

if the file is 4 columns BED file you can set `IsBed = TRUE`

```
> ## loading a 4 columns BED file
> petPath <- system.file("example", "HepG2_centered.bed", package = "R3CPET")
> x <- loadPETs(x, petFile = petPath, IsBed = TRUE, header = FALSE)
```

The `pet(x)` accessor method can be used to read the loaded interactions as *GRanges* object.

- *ChIP-Seq peaks* - All the *ChIP-Seq* peaks of the different TF should be merged into a 4 columns in which the first 3 columns indicate the position of the peak and the last column indicate the associated TF. The `loadTFBS` method can be used to do so.

```
> ## loading a 4 columns BED file
> TFPath <- system.file("example", "HepG2_TF.txt.gz", package = "R3CPET")
> TF <- read.table(TFPath, sep = "\t", header = FALSE)
> head(TF)
##     V1     V2     V3    V4
## 1 chr1 235585 235793 EP300
## 2 chr1 534169 534375 EP300
## 3 chr1 569722 569985 EP300
## 4 chr1 610835 610960 EP300
## 5 chr1 713912 714241 EP300
## 6 chr1 762738 762971 EP300
> x <- loadTFBS(x, tfbsFile = TFPath)
## a total of  135691 binding sites for 6 TF  were loaded
```

- *Protein interaction* - a background `PPI` is needed to do the networks construction. The package comes with two build in `PPI` : the `HPRD` and the `Biogrid PPI`.

```
> data(HPRD)
> data(Biogrid)
>
> PPI.HPRD
## IGRAPH UN-- 9616 39042 --
## + attr: name (v/c)
> PPI.Biogrid
## IGRAPH UN-- 16227 169166 --
## + attr: name (v/c)
```

The `loadPPI` method can be used to load and filter the `PPI` according to different criteria.

```
> loadPPI(object, type = c("HPRD", "Biogrid"), customPPI = NULL,
+     filter = FALSE, term = "GO:0005634", annot = NULL, RPKM = NULL,
+     threshold = 1)
```

if `customPPI` one of the built-in `PPI` will be used otherwise the user can provide a path to an `ncol` formatted graph (two column to indicate interacting nodes) or directly provide an *igraph* object.
The user can also do some filtering to remove proteins that he thinks are not significant. by default the package keeps only the proteins that are located at nucleus (`term = "GO:0005634"`). The user can provide his own annotation to the `annot` parameter. In some cases the user want to keep only the cell specific genes, thus he

can filter genes according to their gene expression by providing a two columns gene expression table to the `RPKM` parameter and set the `threshold`.

```
> ## loading the PPI with GO filtering
> x <- loadPPI(x, type = "HPRD", filter = TRUE)
## loading HPRD network, with 9616 nodes
```

2.2 Creating indexes

When the user thinks that the data that he loaded is ok, he need to create indexes for processing in the further steps. Indexes can be created by calling the method `createIndexes`.

```
> x <- createIndexes(x)

## [1] "Creating PET table"
## [1] "Sorting Table"
## [1] "Get TF associated with each PETs"
## [1] "Creating Motifs table"
## [1] "Sorting Table"
## [1] "Creating hasMotif table"
## [1] "Sorting Table"

> x

## class: ChiapetExperimentData
## 870  interacting regions
## 6 TF used
## Background PPI:
##  nodes: 3447   edges: 14882
## indexes tables have been created
```

2.3 building networks for DNA regions

One all the data are loaded we can go to the next step and build the protein interaction networks for each chromatin loop. The `buildNetworks` is used and a `NetworkCollection` is returned. When the networks are built, the rare and the more frequent edges are removed as they are considered to be not specific. By default, edges that appear in less than 25% or more than 75% of the total networks are removed.

the user can set these values by modifying the `minFreq` and `maxFreq` parameters of the `buildNetworks` method.

```
> nets <- buildNetworks(x, minFreq = 0.1, maxFreq = 0.9)
> nets
```

```
## class NetworkCollection
## 468 network loaded
## 106 diffrent edges has been used
```

The `buildNetworks` uses the *parallel* package to parallelize the processing.If the user has 4 cores, then 4 *R*instances will be lunched at the background each running on a core and handling part of the data.

2.4 Inferring chromatin maintainer networks

At this step the `InferNetworks` can be used to run the HLDA algorithm and infer the set of the most enriched chromatin maintainer networks. by default, the algorithm do a maximum of 500 iteration or stops after 1 hour.

```
InferNetworks(object, thr = 0.5, max_iter = 500L, max_time = 3600L,
    ...)
```

When the algorithm finishes we get a matrix that indicates the degree of partnership of each edge to the inferred networks (each network is distribution over edges). Thus, to get the top elements in each network, we use the parameter `thr` to select the edges that capture `thr`% of the network. Thus, more general networks tend to have bigger number elements and more specific networks have less elements. At the end a `ChromMaintainers` object is returned.

```
> hlda <- InferNetworks(nets)
> hlda
```

```
## class: ChromMaintainers
## HLDA Results:
## ------------
## 145 element have been classified into 10 topics
## Number of different words 128
```

The different slots of the `hlda` object can be accessed using the following accessor methods:

- `topEdges` : to get the list of the top edges in each network.
- `topNodes` : to get the list of the top nodes in each network.
- `networks` : to get a list `igraph` objects.

```
> head(topEdges(hlda))

##      Topic_1         Topic_2          Topic_3
## [1,] "EP300_MYOD1"   "ESR1_ESRRA"     "FOSL2_JUND"
## [2,] "ARNT_EP300"    "ESR1_NCOA1"     "FOSL2_JUN"
## [3,] "GABPA_SP1"     "ESRRA_NCOA1"    "JUN_MYOD1"
## [4,] "CREBBP_SMAD3"  "NCOA1_PPARGC1A" "JUN_SMAD3"
## [5,] "MYOD1_SP1"     "ESR1_PPARGC1A"  "FOSL2_JUNB"
## [6,] "CREBBP_MYOD1"  "ESR1_SMAD3"     ""
##      Topic_4        Topic_5        Topic_6        Topic_7
## [1,] "GABPA_SP1"    "ESR1_JUND"    "JUND_SMAD4"   "FOSL2_JUND"
## [2,] "MAPK1_SP1"    "EP300_ESR1"   "BRCA1_SMAD4"  "EP300_JUN"
## [3,] "MAPK1_SMAD4"  "EP300_SMAD4"  "BCL6_EP300"   "FOSL2_JUN"
## [4,] "MAPK1_SMAD3"  "ESR1_SMAD4"   "ESR1_SMAD3"   "JUN_SMAD3"
## [5,] "JUND_MAPK1"   "EP300_JDP2"   "BRCA1_SMAD3"  ""
## [6,] "BCL6_MAPK1"   ""             ""             ""
##      Topic_8         Topic_9        Topic_10
## [1,] "EP300_GABPA"   "EP300_NCOA1"  "CREBBP_PROX1"
## [2,] "EP300_SP1"     "GTF2B_NCOA1"  "MAPK1_PRKCD"
## [3,] "CREBBP_EP300"  "ESRRA_GTF2B"  "MAPK1_NCOA3"
## [4,] "ATF1_CREBBP"   "ESR1_NCOA3"   "MAPK1_NCOA1"
## [5,] "ATF1_GABPA"    "ESRRA_NCOA3"  ""
## [6,] "CREBBP_GABPA"  "EP300_ESR1"   ""

> head(topNodes(hlda))
```

```
##       Topic_1  Topic_2     Topic_3 Topic_4 Topic_5 Topic_6
## [1,] "EP300"  "ESR1"      "FOSL2" "GABPA" "ESR1"  "JUND"
## [2,] "MYOD1"  "ESRRA"     "JUND"  "SP1"   "JUND"  "SMAD4"
## [3,] "ARNT"   "NCOA1"     "JUN"   "MAPK1" "EP300" "BRCA1"
## [4,] "GABPA"  "PPARGC1A" "MYOD1" "SMAD4" "SMAD4" "BCL6"
## [5,] "SP1"    "SMAD3"     "SMAD3" "SMAD3" "JDP2"  "EP300"
## [6,] "CREBBP" "ARNT"      "JUNB"  "JUND"  ""      "ESR1"
##       Topic_7 Topic_8  Topic_9 Topic_10
## [1,] "FOSL2" "EP300"  "EP300" "CREBBP"
## [2,] "JUND"  "GABPA"  "NCOA1" "PROX1"
## [3,] "EP300" "SP1"    "GTF2B" "MAPK1"
## [4,] "JUN"   "CREBBP" "ESRRA" "PRKCD"
## [5,] "SMAD3" "ATF1"   "ESR1"  "NCOA3"
## [6,] ""      ""       "NCOA3" "NCOA1"
```

The `igraph` networks are not created at the beginning, the `GenerateNetworks` should be used to convert the `topEdges` slot into networks.

```
> hlda <- GenerateNetworks(hlda)
> head(networks(hlda))

## $Network1
## IGRAPH UN-B 7 6 --
## + attr: name (v/c), type (v/c)
##
## $Network2
## IGRAPH UN-B 6 9 --
## + attr: name (v/c), type (v/c)
##
## $Network3
## IGRAPH UN-B 6 5 --
## + attr: name (v/c), type (v/c)
##
## $Network4
## IGRAPH UN-B 11 15 --
## + attr: name (v/c), type (v/c)
##
## $Network5
## IGRAPH UN-B 5 5 --
## + attr: name (v/c), type (v/c)
##
## $Network6
## IGRAPH UN-B 7 5 --
## + attr: name (v/c), type (v/c)
```

if the user wants to annotate each protein in the network by its gene expression he can use the `annotateExpression` method. To use this method the user needs to provide a `data.frame` object, that contains the names of the genes in the first column and their expression value in the second.

```
> data(RPKMS)
> hlda <- annotateExpression(hlda, RPKMS)
> networks(hlda)[[1]]
```

```
## IGRAPH UN-B 7 6 --
## + attr: name (v/c), type (v/c), RPKM (v/n)
```

We can notice that the RPKM attribute was added to the network.

2.5 Cluster DNA interactions by enrichment profile

Till the moment we can say ok, we got the list of out networks and we can do further biological examination to check the results, however,it would be nice if we can know which DNA interactions are enriched for same networks. Thus, the package provide a clustering feature to further analysis.

The `clusterInteractions` can be used to do so. Two types of clustering can be done:

- Supervised : in which the user provides the number of clusters he wants, this part is done using the `sota` method of the *clValid* package.
- non-supervised : in which the number of clusters is determined automatically, performed using the `clues` method of the `clues` package.

The `clusterInteractions` is defined as follow:

```
clusterInteractions(object, method = c("clues", "sota"), nbClus = 20)
```

by default the `clues` method is chosen , if you specify `sota` the default number of clusters is 20.

```
> ## clustering using the 'clues' method
> hlda <- clusterInteractions(hlda, method = "clues")

## clusterInteractions : checking
## clusterInteractions : reading args
## using clues

## DNA interactions have been clustered into 7 cluster
```

2.6 Visualization

The package comes with a bunch of visualization plots to enable the exploration of data mainly through the `plot3CPETRes` method.

```
plot3CPETRes(object, path = "", W = 14, H = 7, type = c("heatmap",
    "clusters", "curve", "avgCurve", "netSim", "networks"), byEdge = TRUE,
    layoutfct = layout.kamada.kawai, ...)
```

To get the genomic coordinates of the regions involved in each cluster the methods `getRegionsIncluster` can be used. To use it the initial initial interaction data should be provided.

```
> getRegionsIncluster(hlda, x, cluster = 3)

## GRanges with 18 ranges and 1 metadata column:
##        seqnames                 ranges strand   |
##           <Rle>              <IRanges>  <Rle>   |
##    [1]     chr1 [  8083072,   8085072]      *   |
##    [2]     chr1 [  8085040,   8087040]      *   |
```

```
##     [3]      chr1 [ 24511310,  24513310]      *   |
##     [4]      chr1 [ 24513094,  24515094]      *   |
##     [5]      chr1 [153916611, 153918611]      *   |
##     ...       ...                     ...     ... ...
##    [14]      chr1 [222762115, 222764115]      *   |
##    [15]      chr1 [244612746, 244614746]      *   |
##    [16]      chr1 [244613896, 244615896]      *   |
##    [17]      chr2 [ 27994738,  27996738]      *   |
##    [18]      chr2 [ 27998645,  28000645]      *   |
##               PET_ID
##          <character>
##     [1]    PET#16.1
##     [2]    PET#16.2
##     [3]    PET#44.1
##     [4]    PET#44.2
##     [5]   PET#181.1
##     ...         ...
##    [14]   PET#230.2
##    [15]   PET#250.1
##    [16]   PET#250.2
##    [17]   PET#288.1
##    [18]   PET#288.2
##    ---
##    seqlengths:
##      chr1 chr10 chr19 chr17 ... chr13  chr7  chr4  chr5
##        NA    NA    NA    NA ...    NA    NA    NA    NA
```

2.6.1 Heatmaps

After clustering the enrichment map can be visualized using the `heatmap` option in the `plot3CPETRes` method. Here each column represent a chromatin-chromatin interaction and each row represents a chromatin maintainer network. The colors indicate the probability that a chromatin-chromatin interaction is maintained by a chromatin maintainer network.

```
> plot3CPETRes(hlda, type = "heatmap")
```

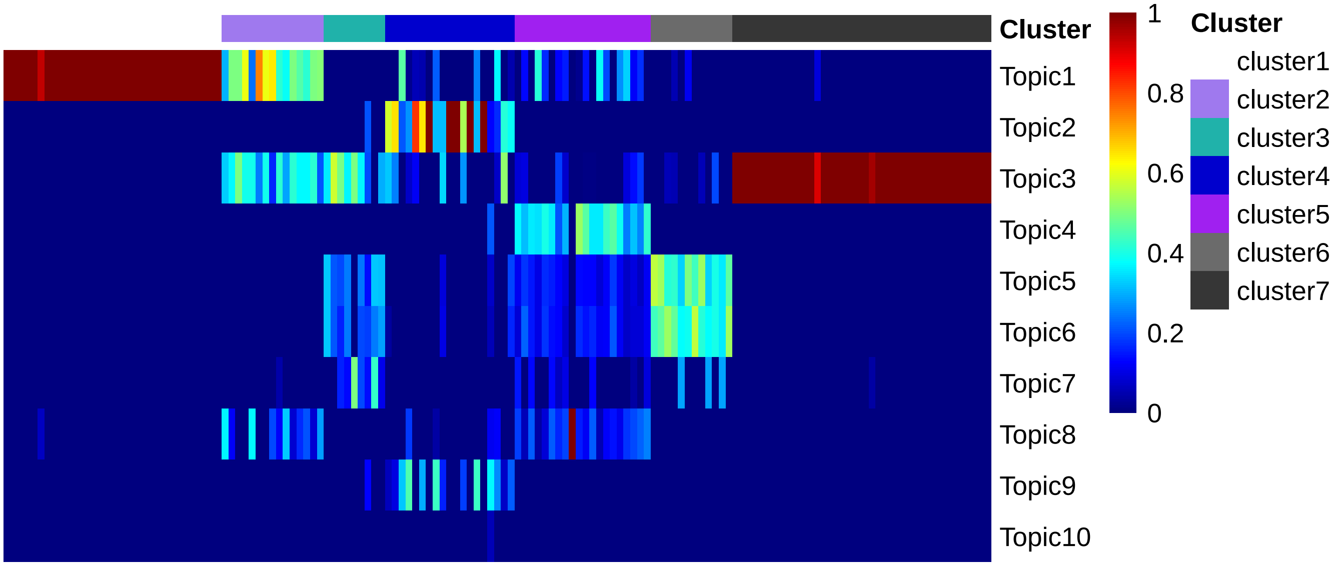

2.6.2 Enrichment curves

An other way to check the enrichment of the different chromatin interactions in the different clusters is by plotting the enrichment and average enrichment curves in of the chromatin interactions in each clusters.

if the `type` parameter of the `plot3CPETRes` method is set to `curve` the enrichment profile of all the interactions per cluster is displayed as shown bellow:

```
> ## plotting curves
> plot3CPETRes(hlda, type = "curve")
```

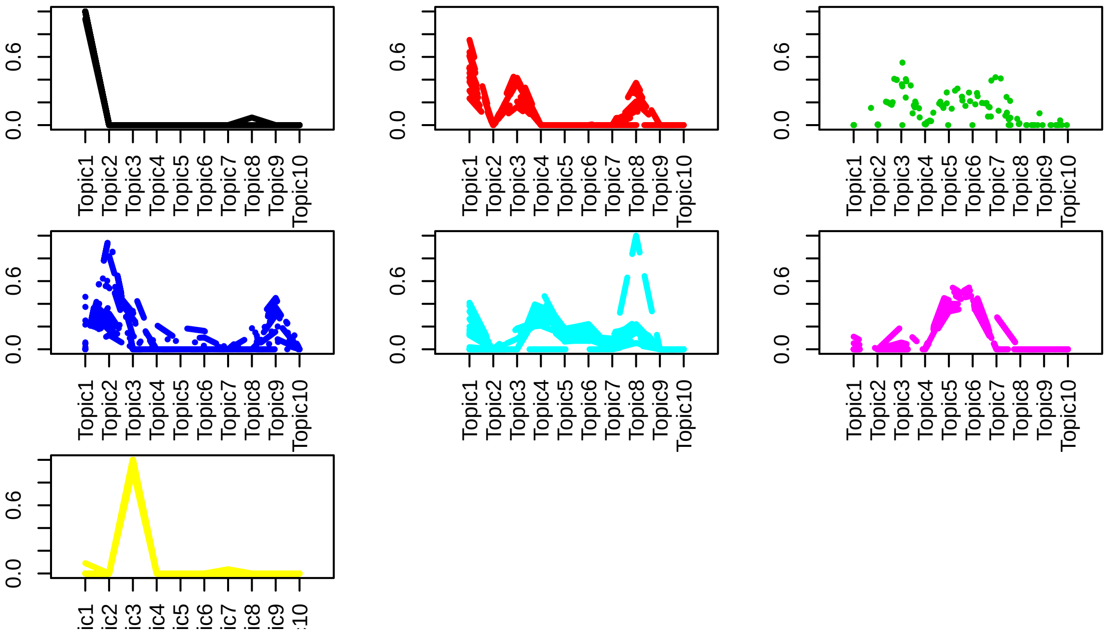

if `type = "avgCurve"` then the average curves are displayed.

```
> ## plotting Average curves
> plot3CPETRes(hlda, type = "avgCurve")
```

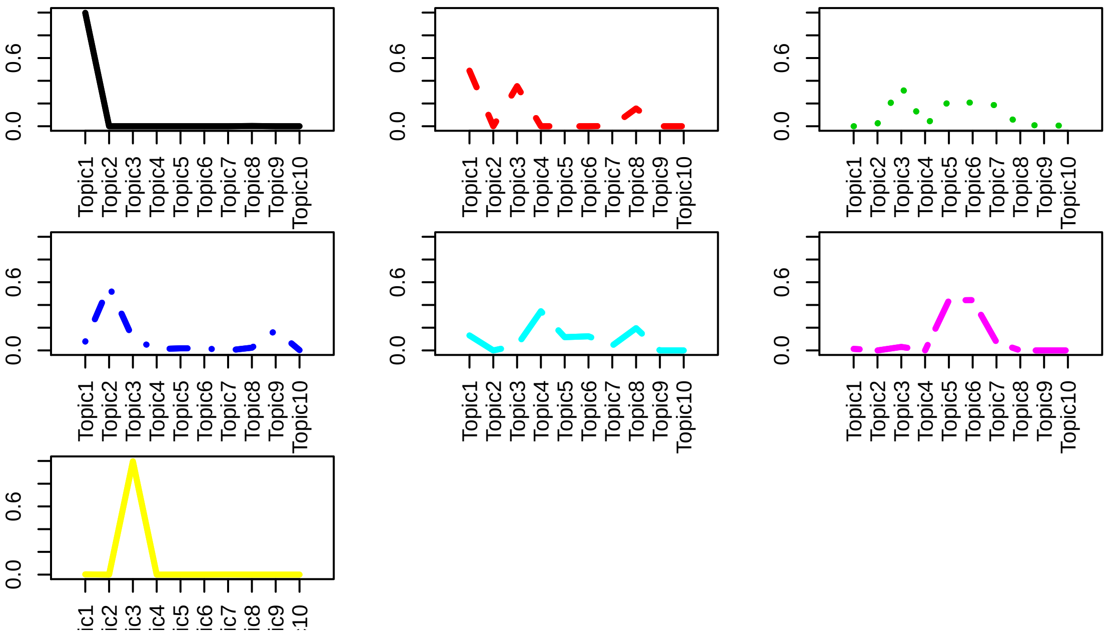

2.6.3 clusters pair-wise scatter plot

In some cases we want to see which two clusters can give a better separation of the data, in this case the `type = "clusters"` option can be used. This option is only available if the clustering have been done using the `clues` method.

```
> ## plotting pair-wise clusters scatter plots
> plot3CPETRes(hlda, type = "clusters")
```

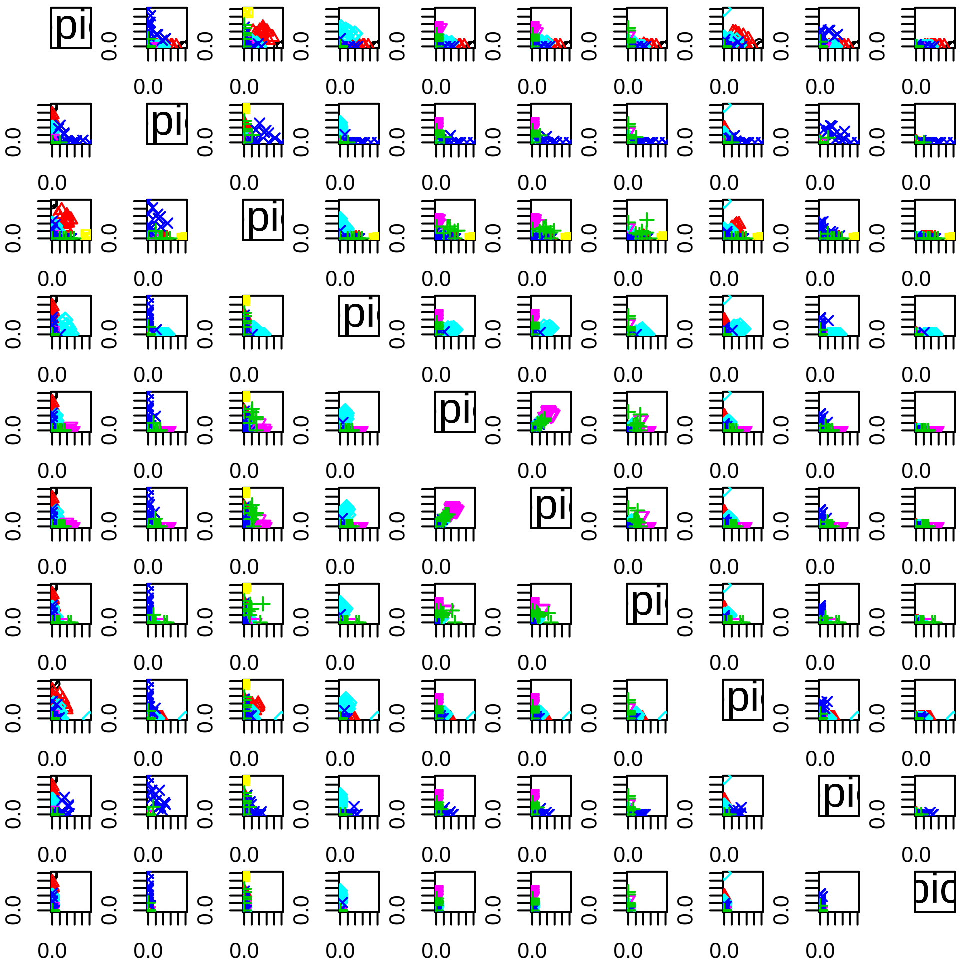

2.6.4 plot networks

if `type ="networks"` is used, a pdf file `AllGraphs.pdf` is created and contains one networks per page. This method returns a `ggplot` list (one for each network). By default the `layout.kamada.kawai` from the *igraph* package is used by the user can pass any other function through the `layoutfct` parameter.

```
> nets_plot <- plot3CPETRes(hlda, type = "networks")
> plot(nets_plot[[4]])
```

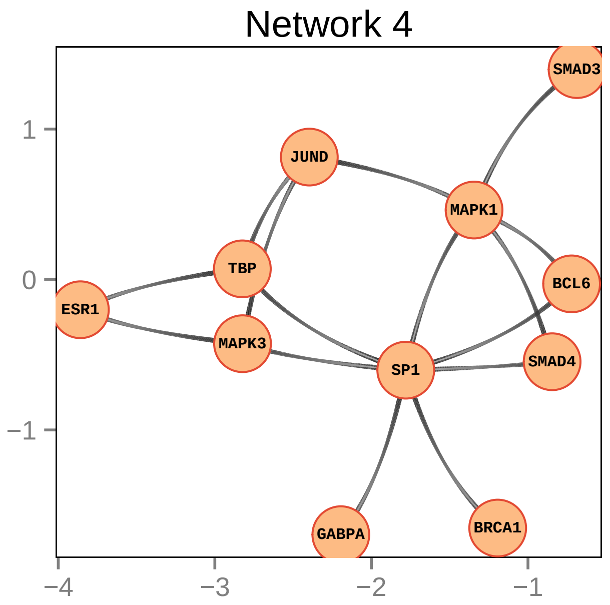

2.6.5 Networks similarity

To the degree to which the infered networks share some common edges we can set `type = "netSim"`. of course, the smaller the similarity the better.

```
> plot3CPETRes(hlda, type = "netSim")
```

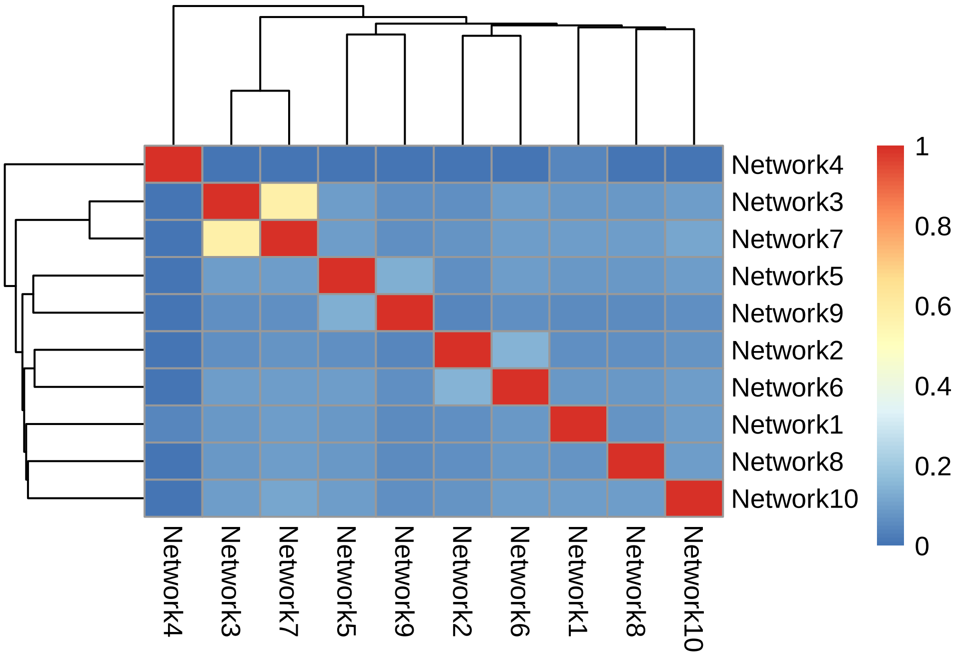

2.6.6 Circos maps

R3CPET also enable the user to plot a basic circos map for a given cluster through the method `visualizeCircos`. The initial data should be passed in the `data` parameter as a `ChiapetExperimentData` object.

```
visualizeCircos(object, data, cluster = 1, chrLenghts = NULL)
```

By default, the human chromosome lengths are used, if the user is using different species he can provide his own chromosome lengths as a two columns `data.frame` that contains the name of the chromosome in the first column and the length in the second one.

```
> visualizeCircos(hlda, x, cluster = 4)
```

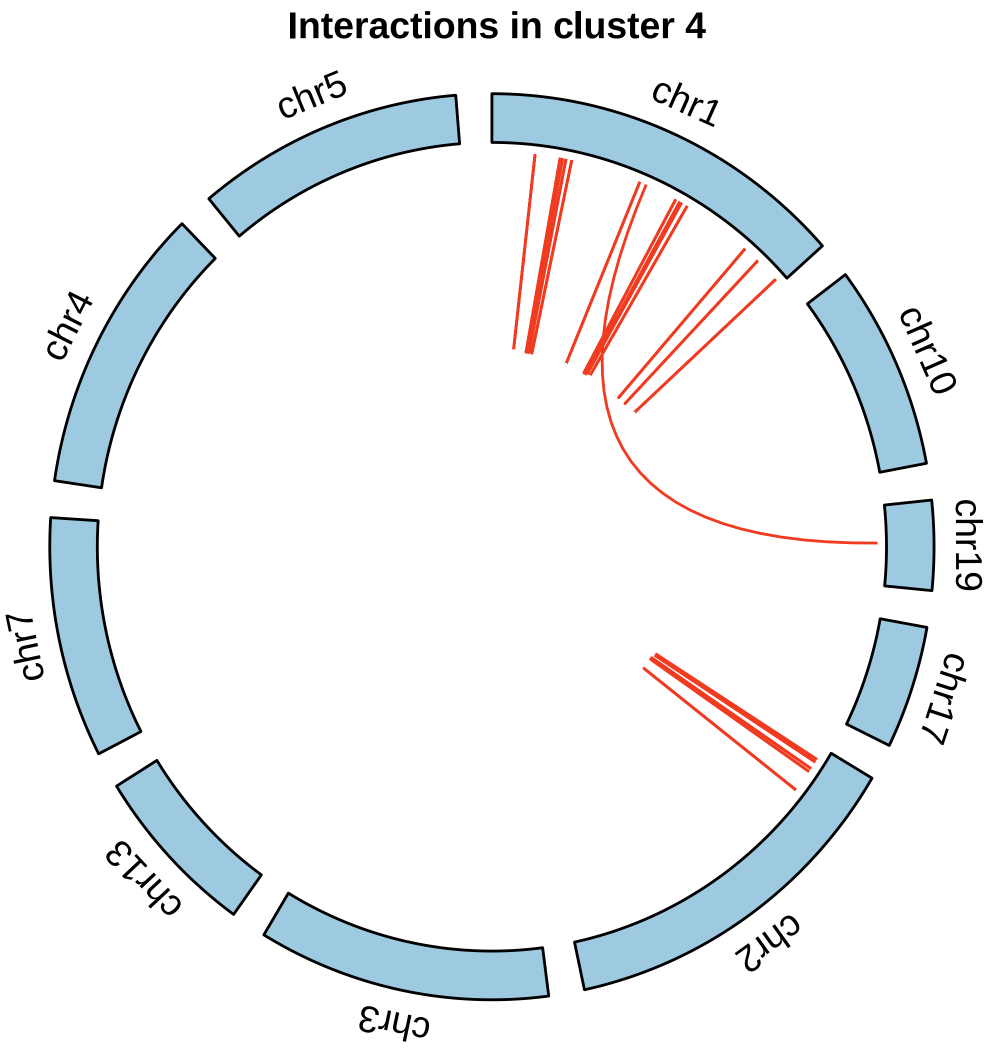

2.7 Gene enrichment

R3CPET enable uses to do a GO analysis using the `DAVID` web service. Two types of enrichment can be done:

- GO enrichment analysis of the proteins of the inferred networks using the `GOEnrich.networks` method.
- GO enrichment of the chromatin interaction clusters using the `GOEnrich.folder` method.

2.7.1 Networks GO enrichment

```
> GOEnrich.networks(object, fdr = 0.05, GOlimit = 5, path = "")
```

The `GOEnrich.networks` method can be used for that. it takes as parameters a `ChromMaintainers` object and a optionally the cu-off `FDR` and the path to save the generated figure. The maximum number of returned GO terms per cluster can be specified by the `GOlimit` parameter.

```
> GOEnrich.networks(hlda, path = ".")
```

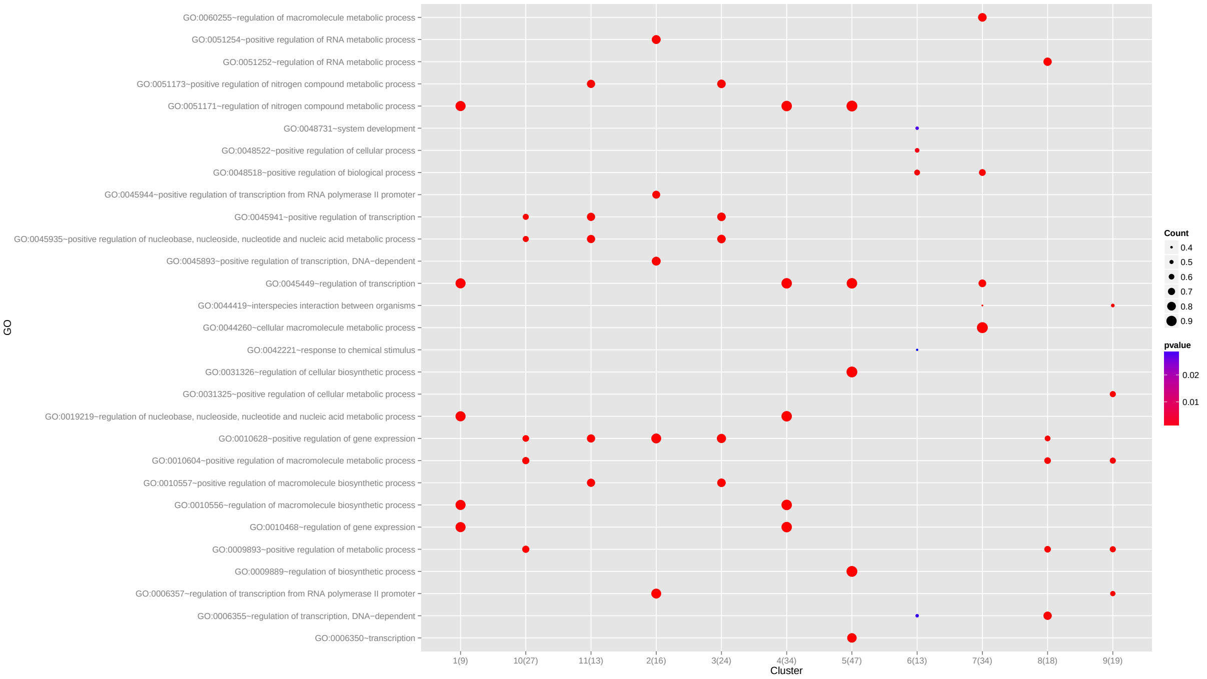

Each column in this plot represent a network and each row represents the GO term to which each network is enriched. The size of the dot is proportional to the number of percentage of the proteins enriched for that term.

2.7.2 GO enrichment of the genes in each cluster

Another question that we want to ask is what are the genes involved in each cluster ? and do they share some specific function. Similarly a GO annotation method is available. However, before doing the GO enrichment the list of list in each each cluster should be extracted, this can be done using the `outputGenesPerClusterToDir` method.

```
outputGenesPerClusterToDir(hdaRes, data, path = "ClustersGenes",
    ...)
```

This function generated a folder (by default named `ClustersGenes`) that contains `.txt` file for each cluster.

```
> outputGenesPerClusterToDir(hlda, x)
```

Then we can use the `GOEnrich.folder` to enrich the list of genes in the created folder.

```
> GOEnrich.folder(folder = "ClustersGenes/")
```

A figure will be generated for the significantly enriched gene lists (example figure bellow).

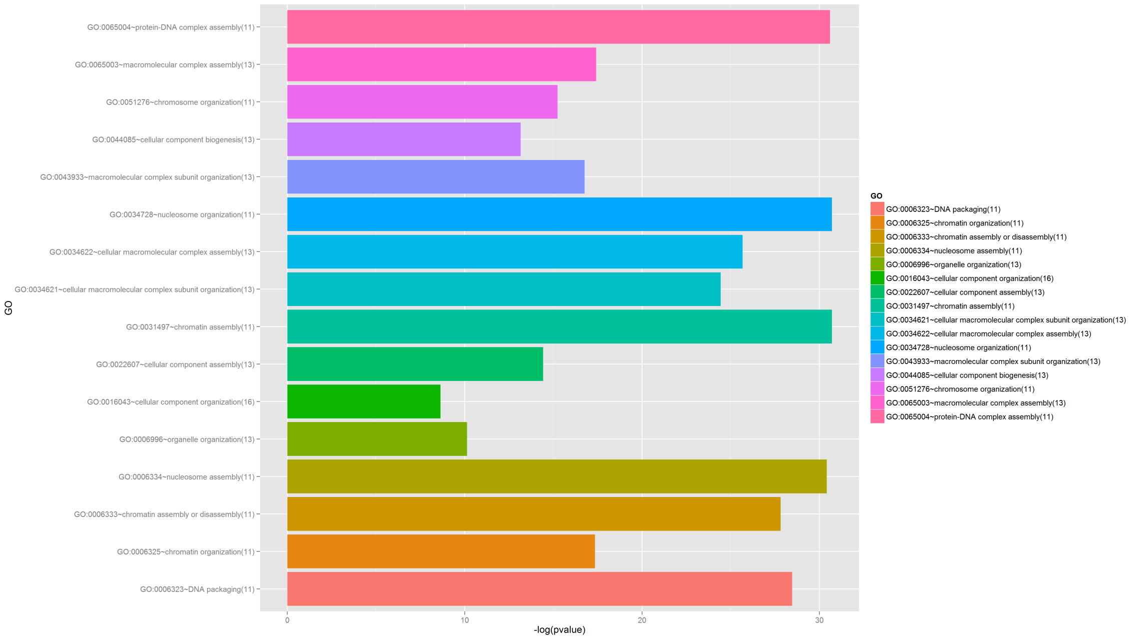

Figure 3: Example of the GO enrichment for a genes of cluster 1

2.8 Using the web interface

After getting all the results (HLDA, clustering, ... etc), the user can display the results using a web browser developped using the *shiny* package. This can be done throght the method `createServer`.

```
createServer(x, nets, hlda)
```

In the website the user can have some information about the raw data, such as the used TF, how many regeions each TF binds, the distribution of interaction per cluster, ... etc. Some of the features are explained in the flowing points

2.8.1 Raw data visualization

Statistics about the 3 types of raw data data (chromatin interactions, TFBS, PPI) can be displayed. Two select options are available under the ”Raw Data” panel:

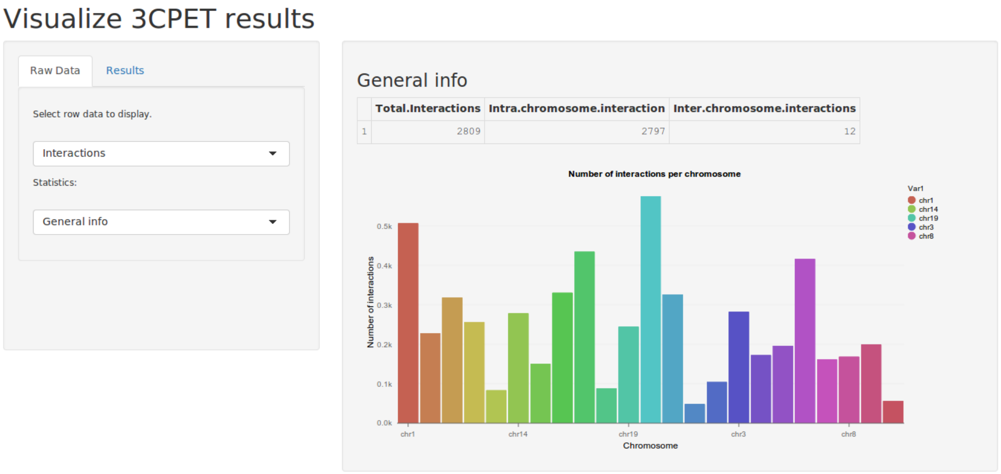

Figure 4: Example showing the histogram of the number of interacting region per chromosome

- data type selection option: in which the user selects the type of data he wants analyze : Interactions, TFBS or PPI.
- statistics selection option : in which the user selects the type of the plot he wants to generate (Figure 4)

2.8.2 Results visualization

This panel also enables the user to interactively analyze his results. Two types of results can be analyzed : The concerning the Chromatin maintainer networks, and the other one about the clustered genomic regions. For example, Figure 5. shows a screenshot in which the user selects a Chromatin maintainer network and display it in an interactive manner using the *D3js* javascript library.

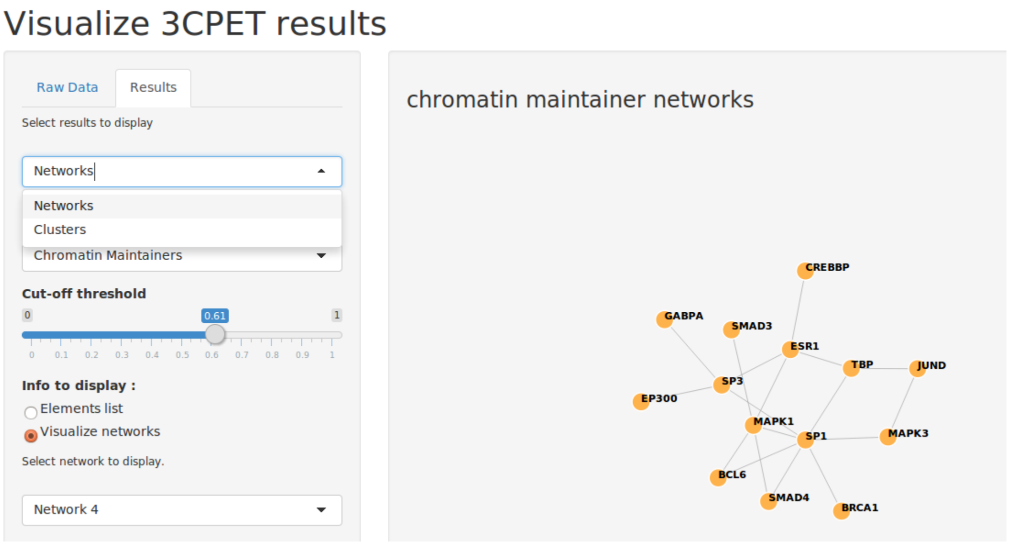

Figure 5: Example of the plots in the web interface

3 Session Info

```
sessionInfo()

## R version 3.1.0 (2014-04-10)
## Platform: x86_64-w64-mingw32/x64 (64-bit)
##
## locale:
## [1] LC_COLLATE=English_United States.1252
## [2] LC_CTYPE=English_United States.1252
## [3] LC_MONETARY=English_United States.1252
## [4] LC_NUMERIC=C
## [5] LC_TIME=English_United States.1252
##
## attached base packages:
## [1] parallel  stats     graphics  grDevices utils
## [6] datasets  methods   base
##
## other attached packages:
## [1] ggplot2_1.0.1         R3CPET_0.99.15
## [3] Rcpp_0.11.5           igraph_0.7.1
## [5] GenomicRanges_1.16.4 GenomeInfoDb_1.0.2
## [7] IRanges_1.22.10       BiocGenerics_0.10.0
## [9] knitr_1.10
##
## loaded via a namespace (and not attached):
##  [1] acepack_1.3-3.3          AnnotationDbi_1.26.1
##  [3] base64enc_0.1-2          BatchJobs_1.6
##  [5] BBmisc_1.9               Biobase_2.24.0
##  [7] BiocParallel_0.6.1       BiocStyle_1.2.0
##  [9] biomaRt_2.20.0           Biostrings_2.32.1
```

```
## [11] biovizBase_1.12.3        bitops_1.0-6
## [13] brew_1.0-6               BSgenome_1.32.0
## [15] checkmate_1.5.2          chron_2.3-45
## [17] class_7.3-10             clues_0.5.6
## [19] cluster_1.15.2           clValid_0.6-6
## [21] codetools_0.2-8          colorspace_1.2-6
## [23] data.table_1.9.4         DAVIDQuery_1.28.0
## [25] DBI_0.3.1                dichromat_2.0-0
## [27] digest_0.6.8             evaluate_0.7
## [29] fail_1.2                 foreach_1.4.2
## [31] foreign_0.8-61           formatR_1.2
## [33] Formula_1.2-1            GenomicAlignments_1.0.6
## [35] GenomicFeatures_1.16.3   ggbio_1.12.10
## [37] grid_3.1.0               gridExtra_0.9.1
## [39] gtable_0.1.2             highr_0.5
## [41] Hmisc_3.15-0             iterators_1.0.7
## [43] labeling_0.3             lattice_0.20-29
## [45] latticeExtra_0.6-26      MASS_7.3-31
## [47] Matrix_1.1-3             munsell_0.4.2
## [49] nnet_7.3-8               pheatmap_1.0.2
## [51] plyr_1.8.2               proto_0.3-10
## [53] RColorBrewer_1.1-2       RCurl_1.95-4.6
## [55] reshape2_1.4.1           rpart_4.1-8
## [57] Rsamtools_1.16.1         RSQLite_1.0.0
## [59] rtracklayer_1.24.2       S4Vectors_0.4.0
## [61] scales_0.2.4             sendmailR_1.2-1
## [63] splines_3.1.0            stats4_3.1.0
## [65] stringr_0.6.2            survival_2.37-7
## [67] tools_3.1.0              VariantAnnotation_1.10.5
## [69] XML_3.98-1.1             XVector_0.4.0
## [71] zlibbioc_1.10.0
```
